# Artesunate in Ferroptosis and Cuproptosis Regulation: Context-Dependent Mechanisms, Interplay, and Therapeutic Implications

**DOI:** 10.3390/ijms27177862

**Published:** 2026-09-02

**Authors:** Kai Feng, Yayi Xia, Jingsheng Liu, Mingxuan Yang

**Affiliations:** The Second Clinical Medical School, Lanzhou University, Lanzhou 730030, China; fengkai0828@163.com (K.F.);

**Keywords:** artesunate, ferroptosis, cuproptosis, iron metabolism, copper metabolism, cancer therapy, neurodegenerative diseases

## Abstract

The discovery of ferroptosis and cuproptosis has significantly expanded the landscape of programmed cell death. Artesunate (ART), a semisynthetic derivative of artemisinin, exhibits pleiotropic pharmacological activities beyond its canonical antimalarial role, including anticancer and neuroprotective effects. However, a comprehensive review systematically integrating ART’s roles in modulating both ferroptosis and cuproptosis remains lacking. This review bridges this gap by synthesizing recent advances. The distinct core mechanisms of ferroptosis and cuproptosis are first outlined. Extensive evidence is then summarized regarding ART-induced ferroptosis in cancers, primarily through disrupting iron homeostasis (e.g., stabilizing transferrin receptor (*TFRC*), promoting ferritinophagy) and impairing antioxidant defenses (e.g., suppressing the System Xc^−^/ glutathione peroxidase 4 (GPX4) axis, targeting peroxiredoxins). In contrast, the anti-cuproptotic effect of ART is currently restricted to Parkinson’s disease (PD) models, mediated by the upregulation of astrocytic metallothionein 2A (MT2A) to chelate excess copper—the only validated cuproptosis-related mechanism of ART to date. The crosstalk between ferroptosis and cuproptosis at shared metabolic nodes, including glutathione depletion, mitochondrial dysfunction, and reactive oxygen species (ROS) amplification, is further delineated as a hypothesis-generating framework. This interplay suggests a theoretical potential for ART—when combined with functional nano-materials—to synchronously engage both death pathways, though direct experimental validation of such dual-pathway synergy for ART as a single agent remains lacking. Finally, translational prospects of ART in cancer therapy, neurodegenerative diseases, and hepatic fibrosis are discussed, together with current challenges and future directions. ART acts as a context-dependent modulator: pro-ferroptotic activity is evident across multiple disease models, whereas anti-cuproptotic activity is currently limited to PD and requires further validation. Systematic characterization of ART’s context-specific effects suggests a preclinical rationale for the future design of ART-based combination strategies targeting metal-dependent cell death, pending further validation.

## 1. Introduction

### 1.1. An Overview of Programmed Cell Death: From Apoptosis to Ferroptosis and Cuproptosis

Programmed cell death (PCD) is a crucial biological process for maintaining tissue homeostasis and eliminating abnormal or redundant cells in multicellular organisms. For a long time, apoptosis, recognized as the classic, non-inflammatory form of PCD, has been extensively studied regarding its molecular mechanisms and physiopathological significance. However, recent research has unveiled a series of novel, precisely regulated cell death modalities have been discovered, expanding our understanding of the mechanisms determining cell fate. Among these, ferroptosis and cuproptosis—driven by dysregulated iron and copper metabolism—have gained attention for their roles in cancer and neurodegenerative diseases [1].

### 1.2. Discovery, Definition, and Physiopathological Significance of Ferroptosis and Cuproptosis

The term “ferroptosis” was first coined in 2012 by the Stockwell laboratory. Its core mechanism involves intracellular ferrous ions (Fe^2+^) catalyzing lipid peroxidation via the Fenton reaction, leading to plasma membrane damage and cell death. It is characterized by the inactivation of antioxidant defense systems such as glutathione peroxidase 4 (GPX4) [2,3,4]. Cuproptosis was formally named in 2022 by Tsvetkov and Golub’s team in *Science*, defined as cell death resulting from the accumulation of excess copper ions within mitochondria, which directly target lipoylated proteins (e.g., dihydrolipoamide S-acetyltransferase (DLAT)) in the tricarboxylic acid (TCA) cycle, triggering proteotoxic stress [5,6,7,8]. These two death modalities are distinct from apoptosis, necroptosis, and pyroptosis in terms of morphology, biochemical features, and regulatory pathways [9,10,11,12]. Studies indicate that ferroptosis and cuproptosis are not only involved in development and homeostasis but also play key roles in the pathogenesis of various diseases. Examples include tumorigenesis [13,14], drug resistance and metastasis [15,16], and neurodegenerative disorders like Parkinson’s and Alzheimer’s diseases [17,18], offering intervention targets for the treatment of related diseases [19].

### 1.3. ART: Evolving from an Antimalarial to a Multi-Effective Agent in Cancer/Nerve Protection

ART, a water-soluble semisynthetic artemisinin derivative, was originally developed as a potent antimalarial agent [20,21]. In recent years, extensive research has uncovered a broad spectrum of pharmacological activities for ART beyond its antimalarial effects [20]. In preclinical oncology models, ART has been shown to inhibit the growth of various solid and hematological tumors through multiple mechanisms, such as reactive oxygen species (ROS) generation, apoptosis induction, angiogenesis inhibition, and cell cycle arrest [22,23,24]. Furthermore, further studies have found that ART can specifically induce ferroptosis in tumor cells by regulating iron metabolism and inhibiting GPX4 activity, revealing a new mechanism for its antitumor action [21,25]. In neurological disease models, ART exhibits neuroprotective effects in Parkinson’s disease models, suggesting potential therapeutic relevance that awaits clinical validation. It can alleviate oxidative stress, inhibit neuroinflammation, promote neuroregeneration, and has been demonstrated in models of Alzheimer’s and Parkinson’s diseases to exert protective effects potentially by modulating metal homeostasis (e.g., upregulating MT2A) [26]. These findings illustrate ART’s transition from an antimalarial to a potential agent for cancer therapy and neuroprotection.

### 1.4. Aim and Framework of This Review

Despite significant individual progress, comprehensive reviews integrating ferroptosis, cuproptosis, and ART are lacking. Key unanswered questions include whether ART can directly regulate cuproptosis and if crosstalk exists between these two pathways under ART treatment. Current evidence for ART-induced ferroptosis is extensive across cancers and fibrotic diseases, whereas evidence for cuproptosis modulation is limited to PD models (astrocytic MT2A); pro-cuproptotic effects are only reported in synthetic nanoplatforms with exogenous copper. This review distinguishes confirmed mechanisms (Level A) from context-specific or hypothetical ones (Level B/C), and frames the latter explicitly as testable hypotheses rather than established therapeutic strategies. To ensure transparency and reduce the risk of selective citation, we searched PubMed, Web of Science, and Scopus for articles published between January 2012 and June 2026 using keyword combinations related to ART (e.g., “ART”, “dihydroartemisinin”), ferroptosis (e.g., “ferroptosis”, “GPX4”, “SLC7A11”), and cuproptosis (e.g., “cuproptosis”, “MT2A”, “DLAT”). Priority was given to peer-reviewed original research articles and systematic reviews. The review elaborates on the independent mechanisms of ferroptosis and cuproptosis (Section 2), summarizes ART-induced ferroptosis (Section 3) and its regulation of cuproptosis (Section 4), discusses crosstalk and synergy (Section 5), assesses preclinical therapeutic implications (Section 6), and outlines challenges and future directions (Section 7).

Recent work by Tolbatov and Marrone [27] established an identical “dual-paradigm” framework for iron/copper-driven regulated cell death, deploying metal dyshomeostasis as a cytotoxic weapon in oncology while harnessing it for neuroprotection in Parkinson’s and Alzheimer’s diseases. That review comprehensively maps nano-ionophore mechanics (e.g., CRISPR-Cas9 RNP@Cu_2_O@SPF, CussOMEp) and the “iron trap” mechanism whereby copper deficiency inactivates multicopper ferroxidases (ceruloplasmin, hephaestin) to trigger ferroptosis, providing a foundational landscape for the field. The present article builds on this work but focuses specifically on ART—an already-marketed antimalarial—as a single molecular node that bidirectionally toggles between ferroptosis induction in tumors (via TFRC/NCOA4/PRDX1) and cuproptosis inhibition in PD (via astrocytic MT2A). By dissecting this context-dependent switch at the level of a clinically familiar scaffold, we complement the broader chelator/nanomedicine perspective laid out by Tolbatov and Marrone and offer a more translationally immediate angle on the iron–copper nexus.

## 2. Molecular Mechanisms of Ferroptosis and Cuproptosis

### 2.1. Core Mechanisms of Ferroptosis

**Figure 1:**
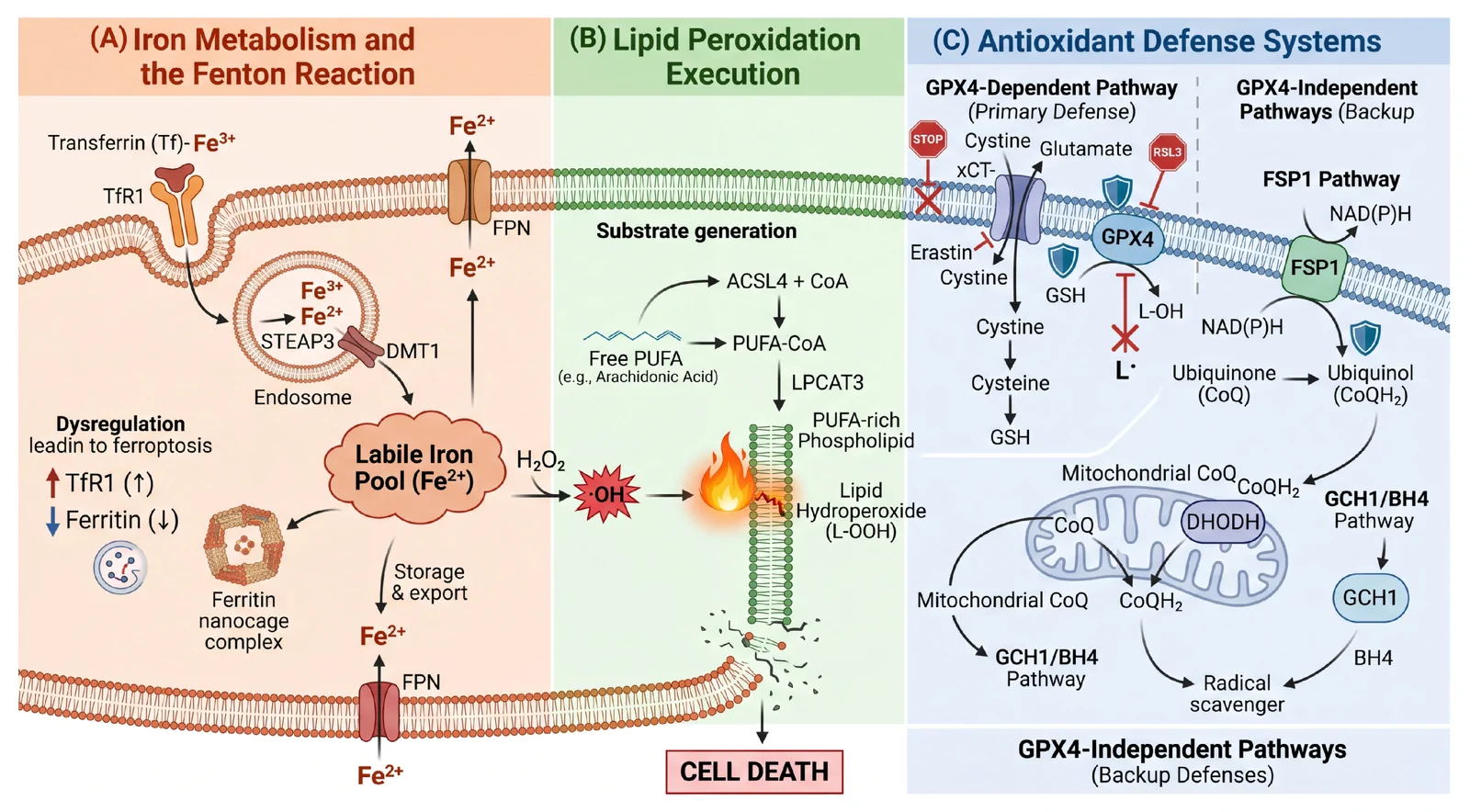
The Core Mechanisms of Ferroptosis: Concerted Actions of Iron Accumulation, Lipid Peroxidation, and Antioxidant Defense Collapse Drive Cell Death. References: [28,35,37,38,39,40,41]. (**A**) Disruption of iron homeostasis and Fenton reaction; (**B**) Lipid peroxidation via ACSL4 and LPCAT3; (**C**) Antioxidant defense system (GPX4 axis and parallel branches). Note: the upward arrow (↑) indicates promotion or increase, the downward arrow (↓) indicates inhibition or decrease, the red crosses (×) indicate blockade or inhibition, the STOP icons indicate termination of the pathway, and the T-bar lines indicate inhibition.

Ferroptosis is an iron-dependent, lipid peroxidation-driven form of regulated cell death [28], as illustrated in Figure 1. Its core machinery centers on three interconnected processes: (i) disruption of iron homeostasis, including increased TFRC-mediated uptake and NCOA4-mediated ferritinophagy [29,30,31,32,33,34]; (ii) enzymatic incorporation of polyunsaturated fatty acids into membrane phospholipids via ACSL4 and LPCAT3 [35,36]; and (iii) collapse of the multilayered antioxidant defense system, dominated by the System Xc^−^/GSH/GPX4 axis [28,37], with parallel branches involving FSP1/CoQH_2_ [38,39], DHODH [40], and GCH1/BH4 [41,42]. However, the relative contribution of each defense branch varies substantially across cell types and contexts; most mechanistic studies rely on genetic knockout models that may not reflect pharmacologically achievable inhibition levels, and the extent to which these pathways operate in parallel under ART treatment remains poorly defined.

### 2.2. Core Mechanisms of Cuproptosis

**Figure 2:**
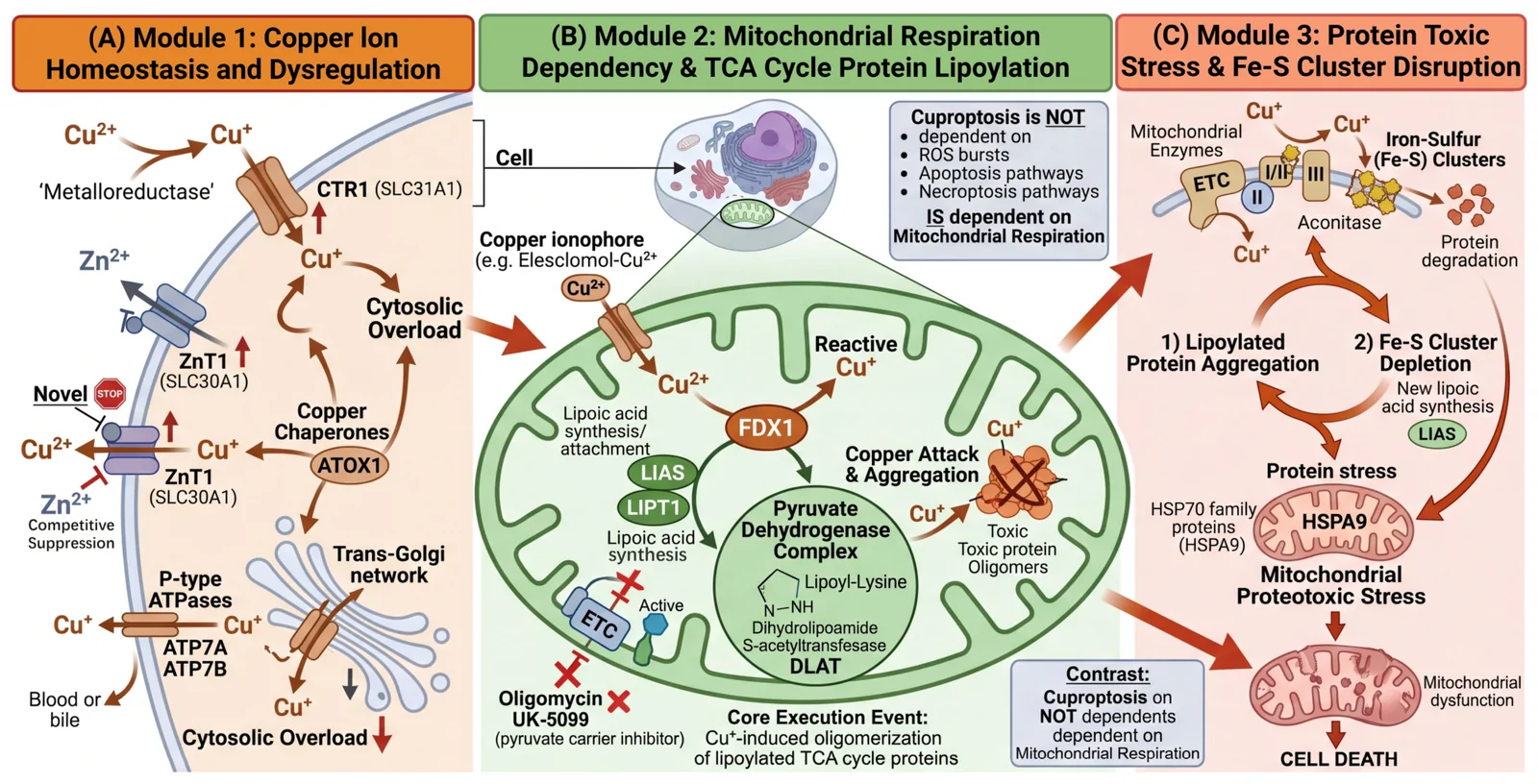
The Core Mechanisms of Cuproptosis: Mitochondrial Copper Overload Triggers Lipoic Acid-Modified Protein Aggregation and Proteotoxic Stress. References: [5,44,46,48]. (**A**) Copper ion homeostasis and dysregulation; (**B**) Mitochondrial respiration dependency and TCA cycle protein lipoylation; (**C**) Protein toxic stress and Fe-S cluster disruption. Note: the upward arrow (↑) indicates promotion or increase, the downward arrow (↓) indicates inhibition or decrease, the red crosses (×) indicate blockade or inhibition, the STOP icons indicate termination of the pathway, and the T-bar lines indicate inhibition.

Cuproptosis is a copper-dependent cell death modality characterized by mitochondrial copper overload, aggregation of lipoylated TCA-cycle proteins, and proteotoxic stress [5], as illustrated in Figure 2. Key regulatory nodes include copper importers CTR1 and ZnT1 [43,44], exporters ATP7A/B [45], the mitochondrial reductase FDX1, which mediates the reduction in Cu^2+^ to Cu^+^ [46], and the lipoylation machinery (LIAS, LIPT1) that modifies proteins such as DLAT [7,47]. Excess mitochondrial Cu^+^ directly binds lipoylated residues, inducing aberrant oligomerization and concurrent destabilization of iron–sulfur cluster proteins, culminating in proteotoxic stress [48,49]. The cuproptosis field remains nascent; the majority of mechanistic data derive from a limited set of cell lines treated with the copper ionophore elesclomol at supraphysiological concentrations. Whether endogenous copper fluctuations without ionophore facilitation can trigger genuine cuproptosis in vivo—and whether ART alone can engage this pathway—remains unaddressed.

### 2.3. Comparisons and Connections Between Ferroptosis and Cuproptosis

**Figure 3:**
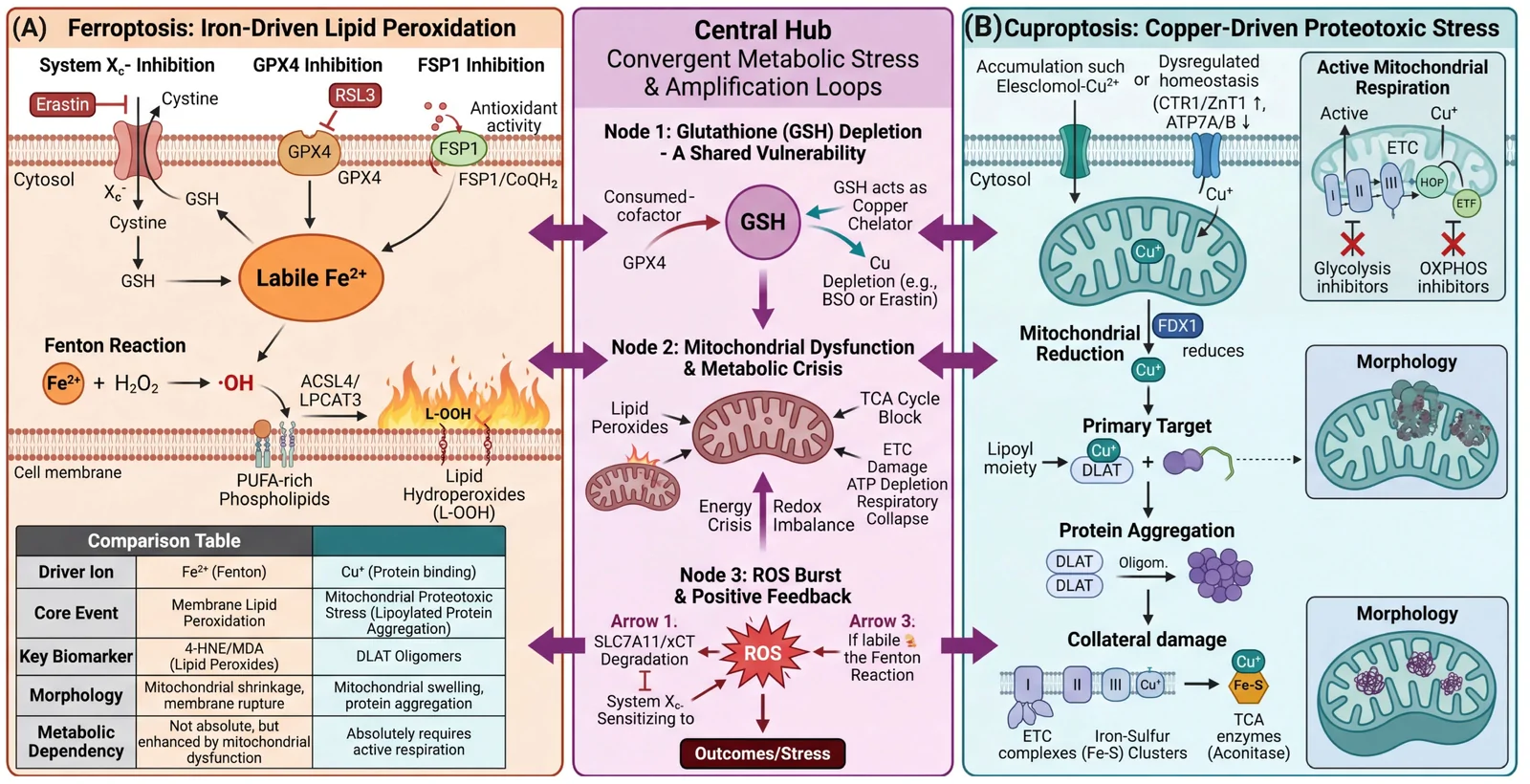
Commonalities and Distinctions Between Ferroptosis and Cuproptosis: From Distinct Driver Ions to Networked Interactions at Shared Metabolic Nodes. References: [5,28,51,52,53]. (**A**) Ferroptosis: iron-driven lipid peroxidation; (**B**) Cuproptosis: copper-driven proteotoxic stress. Note: the upward arrow (↑) indicates promotion or increase, the downward arrow (↓) indicates inhibition or decrease, the red crosses (×) indicate blockade or inhibition, the STOP icons indicate termination of the pathway, and the T-bar lines indicate inhibition.

Ferroptosis and cuproptosis, as two recently discovered metal ion-dependent forms of regulated cell death, clear differences in their driving ions, core execution mechanisms, and morphological features. However, they also share profound interactions and connections at the level of metabolic nodes and cellular stress [50], as illustrated in Figure 3.

#### 2.3.1. Driving Ions (Fe^2+^ vs. Cu^+^) and Triggering Pathways

The fundamental distinction between ferroptosis and cuproptosis lies in the different metal ions they depend on. Ferroptosis is driven by intracellular labile ferrous ions (Fe^2+^) [54]. Excess Fe^2+^ catalyzes the generation of lipid peroxides via the Fenton reaction, which is central to the execution of ferroptotic cell death [28]. Its classic triggering pathways include inhibiting the cystine/glutamate antiporter (System Xc^−^) leading to glutathione depletion, directly inhibiting the activity of glutathione peroxidase 4 (GPX4) [15,37,51], or otherwise impairing cellular antioxidant defenses, such as through inactivation of the FSP1-CoQ_10_ axis [38,39].

In contrast, cuproptosis is driven by excess copper ions (predominantly cuprous ions, Cu^+^, within mitochondria) [5]. Its triggering depends on the accumulation of copper within mitochondria, which is typically facilitated by copper ionophores (e.g., elesclomol) or caused by disrupted copper homeostasis (e.g., CTR1 overexpression or ATP7A/B dysfunction) [55]. Excess Cu^+^ directly targets lipoylated proteins (e.g., DLAT) in the mitochondrial TCA cycle, inducing their oligomerization, and disrupts iron–sulfur cluster proteins, triggering proteotoxic stress, rather than directly catalyzing extensive lipid peroxidation [7,47].

#### 2.3.2. Differences in Morphological and Biochemical Characteristics

Morphologically, ferroptotic cells typically exhibit shrunken mitochondria with increased membrane density, and reduced or absent cristae, eventually accompanied by plasma membrane rupture. Its core biochemical hallmark is the massive accumulation of peroxidation products (e.g., MDA, 4-HNE) derived from polyunsaturated fatty acid phospholipids on the cell membrane [28,37,54].

The morphological features of cuproptosis are still under investigation, but current evidence indicates it does not cause the typical ferroptosis-like mitochondrial shrinkage. Its core biochemical signature is the abnormal oligomeric aggregation of lipoylated proteins (e.g., DLAT) and the degradation of iron–sulfur cluster proteins within mitochondria. Furthermore, cuproptosis strictly depends on active mitochondrial respiration and oxidative phosphorylation, whereas the occurrence of ferroptosis, while potentially exacerbated by mitochondrial dysfunction, is not absolutely dependent on it [5,7,47].

#### 2.3.3. Shared Metabolic Nodes (GSH Depletion, Mitochondrial Dysfunction, ROS Burst)

Despite their distinct mechanisms, ferroptosis and cuproptosis intersect at several key metabolic nodes, including glutathione (GSH) depletion, mitochondrial dysfunction, and reactive oxygen species (ROS) accumulation.

Glutathione Depletion: GSH is a key molecule linking the two pathways. In ferroptosis, as an essential cofactor for GPX4, GSH depletion directly leads to GPX4 inactivation and uncontrolled lipid peroxidation [51,56]. In cuproptosis, as a major intracellular copper chelator, GSH depletion releases free copper ions, promoting copper accumulation and toxicity within mitochondria [57,58]. Strategies depleting GSH (e.g., using BSO or erastin) can simultaneously sensitize cells to both death modalities.

Mitochondrial Dysfunction and the TCA Cycle: Mitochondria are a central site for their interaction. Cuproptosis directly disrupts the TCA cycle (via DLAT aggregation) and the electron transport chain (via iron–sulfur cluster protein degradation), leading to mitochondrial functional collapse and impaired ATP production [5]. Such mitochondrial damage and the ensuing energy crisis exacerbate cellular redox imbalance, potentially fostering a lipid peroxidation-prone microenvironment conducive to ferroptosis [15]. Conversely, lipid peroxides and ROS generated during ferroptosis can also damage mitochondria, potentially affecting their metabolic state and thereby influencing cellular sensitivity to cuproptosis.

Reactive Oxygen Species Burst: Although cuproptosis was initially considered independent of a massive ROS burst, mitochondrial dysfunction and electron transport chain collapse inevitably lead to increased ROS production [57]. These ROS can further deplete antioxidants (like GSH) and may exacerbate lipid peroxidation via the Fenton reaction (if free iron is present), thus forming a positive feedback loop with the ferroptosis pathway. Studies indicate that copper overload can induce ferroptosis by causing SLC7A11 degradation through ROS production [52,59].

In summary, ferroptosis and cuproptosis represent two distinct death programs driven by different metal ions with unique molecular signatures. However, they are intricately linked through shared nodes such as the GSH pool, mitochondrial metabolism, and redox homeostasis [60]. This interplay suggests the hypothetical possibility that interventions targeting one pathway may influence the other. Whether such crosstalk can be leveraged to develop clinically meaningful synergistic anti-cancer strategies via ART remains an open question requiring systematic experimental validation; this review therefore positions ART-related crosstalk hypotheses (Table 1) as starting points for future investigation rather than established therapeutic paradigms. However, several caveats should be noted. First, the majority of evidence for shared metabolic nodes (GSH depletion, mitochondrial dysfunction, ROS burst) derives from studies using pharmacological agents other than ART (e.g., erastin, BSO, elesclomol). Whether ART engages these nodes with comparable kinetics and magnitude remains to be systematically tested. Second, the proposed bidirectional amplification between ferroptosis and cuproptosis (e.g., cuproptosis-driven Fe–S cluster degradation fueling ferroptosis) has been demonstrated only in synthetic nanoplatforms with supraphysiological metal concentrations; its occurrence under physiological conditions is unconfirmed. Third, cell-type specificity is a major confounder: the same GSH-depleting stimulus can promote ferroptosis in cancer cells while protecting astrocytes from cuproptosis, depending on the dominant stress-response pathway. These considerations underscore the need for ART-specific validation of each proposed crosstalk mechanism, as classified in Table 1.

## 3. Mechanisms of ART-Induced Ferroptosis

### 3.1. Chemical Properties and Pharmacological Basis of ART

As noted previously, the endoperoxide bridge (-O-O-) of ART is essential for its pharmacological activity. Throughout this review, ‘ART’ refers specifically to artesunate. Dihydroartemisinin (DHA, the active metabolite of ART formed by rapid esterase-mediated hydrolysis), the parent sesquiterpene lactone artemisinin, and other synthetic artemisinin derivatives (e.g., artemether, arteether) are discussed under their own designations. These molecules differ substantially in pharmacokinetics, bioavailability, and biological activity; therefore, findings from derivative-only studies are not extrapolated to ART without direct experimental confirmation. In vivo, ART is rapidly hydrolyzed to its active metabolite dihydroartemisinin (DHA). ART’s antimalarial activity relies on iron-dependent cleavage of its endoperoxide bridge, generating reactive radicals that kill the parasite [20]. This iron-dependent radical generation also underlies ART’s ability to induce oxidative stress-related cell death (e.g., ferroptosis) in cancer cells. Beyond antimalarial use, ART exhibits broad anti-tumor activity in preclinical models through multiple mechanisms, including cell cycle arrest, angiogenesis inhibition, and induction of apoptosis, autophagy, and ferroptosis [61,68,69]. Tumor cells, with their higher iron demand and oxidative stress levels, are preferentially sensitive to ART-mediated oxidative damage, making ART a candidate ferroptosis inducer [64]. However, the extent to which ART’s antimalarial mechanism (iron-dependent radical generation) translates directly to its ferroptosis-inducing activity in mammalian cells remains incompletely defined, as intracellular iron speciation and compartmentalization differ substantially from those in Plasmodium parasites.

Throughout this section, it is important to distinguish ferroptosis-associated biochemical changes—such as altered ROS, GSH, GPX4, labile iron, or lipid peroxidation—from experimentally demonstrated ferroptotic cell death. According to established criteria, ferroptosis should be confirmed by suppression of cell death via both an iron chelator (e.g., deferoxamine) and a lipophilic antioxidant (e.g., ferrostatin-1, liproxstatin-1), alongside accumulation of lipid hydroperoxides. Given that ART can concurrently induce apoptosis and autophagy, pharmacological or genetic rescue experiments are essential to attribute observed cell death specifically to ferroptosis. Studies summarized below are annotated accordingly [54].

### 3.2. Targeting Iron Metabolism-Related Proteins

**Figure 4:**
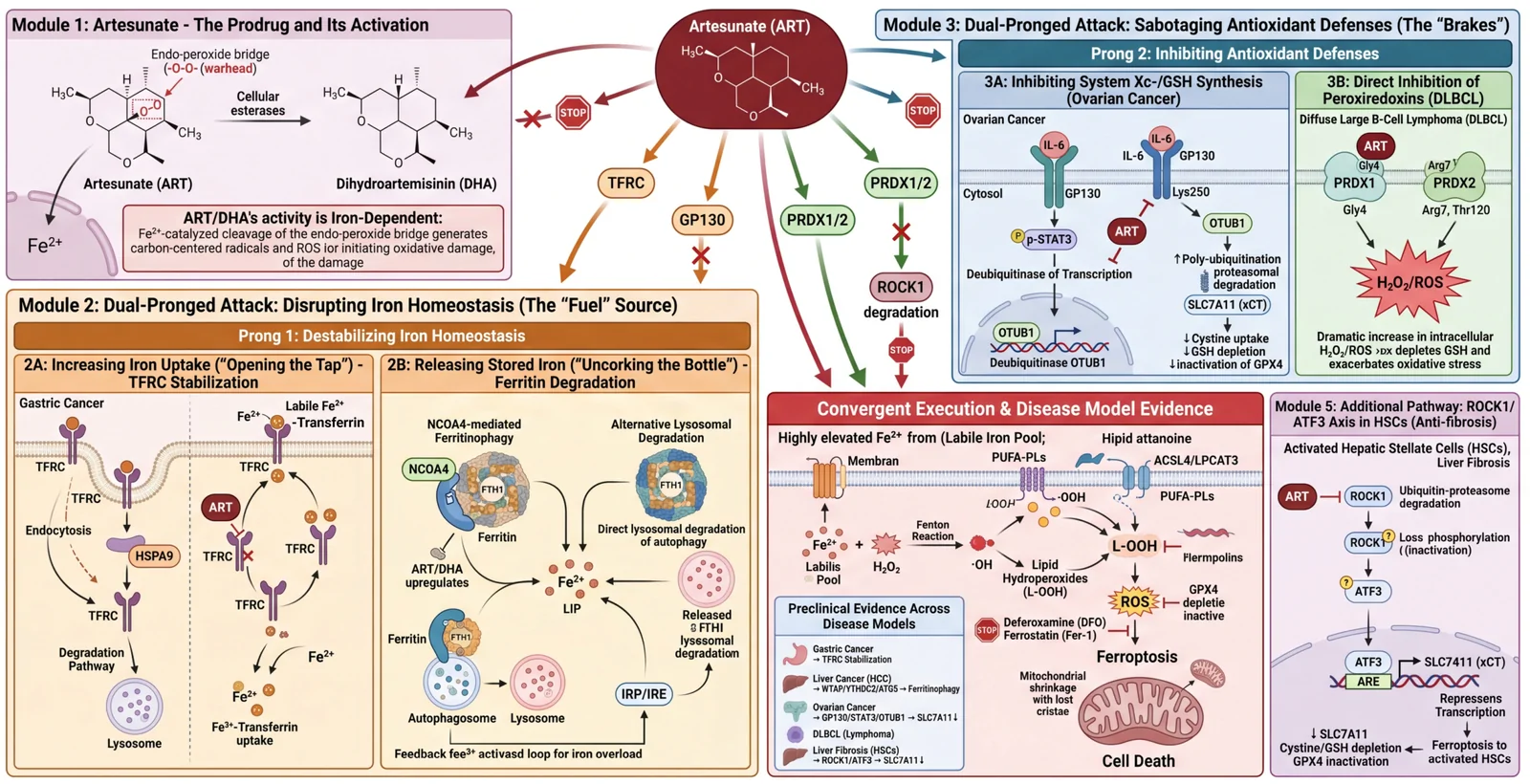
The Multi-Target and Multi-Layered Molecular Mechanisms of ART in Driving Ferroptosis. References: [61,62,63,64,69]. Note: the upward arrow (↑) indicates promotion or increase, the downward arrow (↓) indicates inhibition or decrease, the red crosses (×) indicate blockade or inhibition, the STOP icons indicate termination of the pathway, and the T-bar lines indicate inhibition.

One of the core mechanisms by which ART induces ferroptosis is the disruption of intracellular iron homeostasis by directly or indirectly regulating key iron metabolism proteins, leading to an abnormal increase in the labile iron pool (LIP) level, thereby driving the Fenton reaction and lipid peroxidation, as illustrated in Figure 4.

#### 3.2.1. Binding TFRC and Blocking Its Interaction with HSPA9, Increasing Iron Uptake (Gastric Cancer)

Transferrin receptor 1 (TFRC) is the primary mediator of cellular iron uptake. In gastric cancer cells, ART directly binds TFRC with high affinity, disrupting its interaction with heat shock protein HSPA9 and preventing lysosomal degradation [64]. This stabilizes TFRC on the cell membrane, prolongs its half-life, and sustains transferrin-iron endocytosis, sharply elevating intracellular Fe^2+^ levels. Knocking down TFRC attenuated ART-induced iron accumulation, lipid peroxidation, and cell death, while overexpression enhanced these effects. However, this study predominantly relied on genetic validation (TFRC knockdown/overexpression) and biochemical ferroptosis-associated markers; direct pharmacological rescue with ferrostatin-1 or liproxstatin-1 was not reported. Thus, while the evidence strongly implicates a ferroptotic mechanism, the study demonstrates ferroptosis-associated phenotypic changes with genetic—rather than pharmacological—validation of cell death modality. This mechanism was validated in a gastric cancer xenograft model, where ART inhibited tumor growth with increased iron content and lipid peroxidation markers. While this TFRC-stabilization mechanism is well-supported in gastric cancer, its relevance in other cancer types with lower TFRC expression or alternative iron acquisition pathways (e.g., LIP1-mediated uptake) has not been examined.

#### 3.2.2. Activating Lysosome-Mediated Ferritin Degradation, Elevating Intracellular Free Iron Levels

ART and DHA also amplify iron overload by promoting ferritin degradation via NCOA4-mediated ferritinophagy [70]. ART upregulates NCOA4 expression, enhancing binding of ferritin heavy chain (FTH1) to NCOA4 and targeting ferritin to autolysosomes for degradation [66]. In osteosarcoma and ovarian serous carcinoma cells, ART induces ferroptosis through NCOA4-dependent ferritinophagy; knocking down NCOA4 inhibits this process [65]. DHA-induced ferritin degradation may also occur independently of classical autophagy, rapidly increasing free iron and sensitizing cells to GPX4 inhibitors. Released free iron can bind IRPs, upregulating TFRC via the IRP/IRE system and creating a positive feedback loop that worsens iron overload [71]. Studies in hepatic stellate cells and fibroblasts further confirm that ART/DHA induces ferroptosis-associated cell death via ferritinophagy, exerting anti-fibrotic effects. In osteosarcoma, NCOA4 silencing, together with ferrostatin-1 and deferoxamine, reversed ART-induced lipid peroxidation and cell death, providing pharmacological validation of ferroptotic cell death. By contrast, the hepatic stellate cell studies primarily employed genetic approaches (ROCK1/ATF3 modulation) to infer ferroptosis; pharmacological rescue experiments in this context remain to be directly demonstrated [69].

In summary, ART employs a dual strategy of enhancing iron uptake (TFRC stabilization) and releasing stored iron (ferritinophagy) to disrupt iron homeostasis. While these two strategies are conceptually complementary, it remains unclear whether they operate additively or synergistically within the same cell. Furthermore, the relative reliance on TFRC stabilization versus NCOA4-mediated ferritinophagy appears cell-type dependent; a systematic comparison across cancer lineages is lacking.

### 3.3. Regulating the Antioxidant Defense System

Another core strategy for ART to induce ferroptosis, besides disrupting iron homeostasis, is to directly impair the cellular antioxidant defense system, particularly the GSH synthesis pathway and peroxide scavenging systems, thereby weakening the cell’s ability to resist lipid peroxidation, as shown in Figure 4.

#### 3.3.1. Inhibiting the System Xc^−^/GSH/GPX4 Axis (Downregulating SLC7A11 via GP130/IL-6/STAT3/OTUB1 in Ovarian Cancer)

System Xc^−^ imports cystine for GSH synthesis. Its functional subunit SLC7A11 (xCT) determines intracellular GSH levels and GPX4 activity. In ovarian cancer cells, ART inhibits SLC7A11 expression through a signaling axis involving GP130/IL-6/STAT3/OTUB1 [63]. ART binds to lysine 250 of GP130, disrupting the IL-6/IL-6R/GP130 complex, inhibiting STAT3 phosphorylation, and reducing OTUB1 transcription. Lower OTUB1 decreases deubiquitination of SLC7A11, leading to its proteasomal degradation. Downregulation of SLC7A11 limits cystine uptake, depletes GSH, inactivates GPX4, and triggers ferroptosis. This mechanism was validated both in vitro and in vivo, revealing GP130 as a direct target of ART. However, the study established SLC7A11 downregulation, GSH depletion, and GPX4 inactivation as ferroptosis-associated biochemical events; explicit rescue with ferrostatin-1, liproxstatin-1, or iron chelation to confirm ferroptotic cell death was not performed. Related ART studies in ovarian serous carcinoma cells demonstrated that ART-induced cell death and lipid peroxidation were reversed by both deferoxamine and ferrostatin-1, confirming ferroptotic cell death in this cellular context [72].

#### 3.3.2. Directly Targeting and Inhibiting Peroxiredoxins PRDX1/PRDX2 (Diffuse Large B-Cell Lymphoma)

In diffuse large B-cell lymphoma (DLBCL), ART directly targets and inhibits PRDX1 and PRDX2, inducing ferroptosis [61]. Using small-molecule probe pull-down coupled with mass spectrometry, molecular docking, and point mutation verification, ART was found to bind Gly^4^ of PRDX1 and Arg7/Thr120 of PRDX2, inhibiting their peroxidase activity and elevating intracellular ROS. Elevated ROS depletes GSH and synergizes with iron to exacerbate lipid peroxidation. Knocking down PRDX1/2 mimicked ART-induced ferroptosis, while PRDX2 overexpression attenuated it. This genetic rescue validates PRDX1/2 as ferroptosis-relevant targets in DLBCL; however, the study did not employ pharmacological ferroptosis inhibitors (ferrostatin-1, liproxstatin-1) or iron chelation as orthogonal validation, and the possibility that concurrent apoptotic or autophagic cell death contributes to the observed phenotype cannot be entirely excluded. Consistent with the lower expression of PRDX1/2 in normal cells, ART exhibited selective cytotoxicity towards DLBCL cells with minimal organ toxicity in a xenograft model. This identifies PRDX1/2 as potential targets for DLBCL treatment. However, the generalizability of this finding to other lymphoma subtypes or solid tumors has not been tested. Moreover, the extent to which ART-mediated GPX4 inhibition (via SLC7A11 downregulation) versus PRDX inhibition contributes to overall ferroptosis execution in a given context remains unresolved, as most studies examine only one pathway in isolation.

### 3.4. Other Pathways: Regulating the ROCK1/ATF3 Axis to Promote HSC Ferroptosis (Anti-Hepatic Fibrosis)

ART also induces ferroptosis in activated hepatic stellate cells (HSCs) via the ROCK1/ATF3/SLC7A11 axis, exerting anti-fibrotic effects [28,62]. ART promotes ubiquitination and degradation of ROCK1, relieving its inhibitory phosphorylation of ATF3. Dephosphorylated ATF3 accumulates in the nucleus and binds the antioxidant response element (ARE), inhibiting SLC7A11 transcription. Downregulation of SLC7A11 depletes GSH, inactivates GPX4, and triggers HSC ferroptosis. In a CCl_4_-induced mouse fibrosis model, ART improved liver pathology, reduced collagen deposition, and increased iron deposition; interfering with Atf3 or overexpressing Rock1 in HSCs reversed these effects. This study establishes a non-tumor indication for ART-induced ferroptosis-associated cell death. The conclusions rest on genetic manipulation of the ROCK1/ATF3 axis and biochemical evidence (SLC7A11 downregulation, GSH depletion, iron deposition); pharmacological rescue with ferrostatin-1 or liproxstatin-1 was not explicitly demonstrated in this model. Thus, the ferroptotic nature of HSC death is inferred from genetic and biochemical correlates, which—while strongly supportive—do not constitute the full criterion of demonstrated ferroptotic cell death. However, the specificity of the ROCK1/ATF3 axis to activated versus quiescent HSCs or hepatocytes warrants further validation, as off-target ferroptosis in parenchymal cells could exacerbate liver injury.

### 3.5. Evidence of ART-Induced Ferroptosis in Disease Models

ART-induced ferroptosis-associated cell death has been reported in preclinical models of gastric cancer, liver cancer, lymphoma, ovarian cancer, osteosarcoma, and acute myeloid leukemia, with varying tiers of validation—ranging from orthogonal pharmacological rescue (e.g., ferrostatin-1, liproxstatin-1, deferoxamine) and genetic approaches to biochemical phenotyping alone [66,73,74]. In gastric cancer, ART stabilizes TFRC to increase iron uptake [64]. In hepatocellular carcinoma, ART upregulates WTAP-mediated m^6^A methylation of ATG5 mRNA, enhancing autophagy-dependent ferritin degradation and ferroptosis [66]. In DLBCL, ART directly inhibits PRDX1/2 [61]. In ovarian cancer, it suppresses SLC7A11 via the GP130/STAT3/OTUB1 axis [63]. In osteosarcoma, it activates NCOA4-mediated ferritinophagy [65]. A comprehensive summary of these key studies, including ART doses, molecular targets, and experimental limitations, is provided in Table 2. Notably, among the studies cited above, artesunate-induced ferroptosis has been orthogonally validated by pharmacological rescue with liproxstatin-1, ferrostatin-1, and desferoxamine in Burkitt’s lymphoma cells, and by ferrostatin-1 (with additional reversal by deferoxamine) in KRAS-mutant pancreatic cancer cells; the remaining cited studies vary in the extent of pharmacological versus genetic validation, as detailed in Table 2.

Although the collective evidence supports a broad pro-ferroptotic role for ART across multiple disease models, several caveats deserve mention. First, most studies employ ART concentrations that exceed clinically achievable plasma levels. Second, the majority of in vivo models are subcutaneous xenografts, which do not recapitulate the tumor microenvironment or metastatic niches. Third, direct head-to-head comparisons of ART’s ferroptosis potency versus established ferroptosis inducers (e.g., erastin, RSL3) are rarely performed, limiting quantitative benchmarking.

## 4. Evidence and Mechanisms of ART in Regulating Cuproptosis

Unlike its well-documented, standalone role in inducing ferroptosis across multiple oncology and fibrotic disease models, evidence for ART’s regulation of cuproptosis remains sparse and highly context-restricted. To date: (1) the only validated standalone anti-cuproptosis mechanism links ART to PD models via the astrocytic MT2A axis; this validation is achieved through MT2A knockdown rescue experiments that abolish ART’s neuroprotective effects, establishing a causal link between ART→MT2A→cuproptosis-associated protein modulation→dopaminergic neuron survival [26]—though direct biochemical demonstration of DLAT oligomerization reversal or Fe-S cluster restoration by ART remains to be performed; (2) no direct evidence confirms ART modulates core cuproptosis execution proteins (e.g., DLAT, FDX1) in cancer or other non-neurodegenerative contexts as a single agent; (3) pro-cuproptotic correlations of ART emerge only in co-delivery systems with copper-based nanomaterials. In these engineered platforms, cuproptosis is primarily driven by nanomaterial-released Cu^2+^ under supraphysiological conditions; ART functions as a ‘cuproptosis sensitizer’ via GSH depletion rather than a direct ‘cuproptosis inducer.’ Importantly, the parent artemisinin, DHA, and ART are chemically distinct and cannot be used interchangeably: ART-alone induction of cuproptosis has not been demonstrated in any cell type to date. A thorough search of the 2026 literature confirms this boundary remains valid [75]. This chapter therefore presents confirmed PD-specific findings separately from hypothetical/generalizable mechanisms.

### 4.1. Anti-Cuproptosis Effect of ART in Parkinson’s Disease Models

Copper dyshomeostasis is implicated in the pathogenesis of Parkinson’s disease, and excess copper can trigger cuproptosis in dopaminergic neurons. ART protects neurons by targeting astrocytes to remodel copper homeostasis.

#### 4.1.1. Targeting Astrocytic MT2A and Upregulating Its Expression

A study using a 6-hydroxydopamine (6-OHDA)-induced PD rat model found that ART significantly improved motor dysfunction and protected dopaminergic neurons in the substantia nigra pars compacta [26]. By constructing co-culture systems of neurons, neuron-microglia, and neuron-astrocytes, the researchers discovered that the neuroprotective effect of ART strictly depended on the presence of astrocytes, suggesting its cellular target resides in astrocytes. To identify the direct molecular target, the research team performed high-throughput screening using the HuProt™ 20K human proteome microarray, identifying 867 potential ART-binding proteins from approximately 20,000 proteins. Bioinformatics analysis revealed these proteins were significantly enriched in pathways related to metal ion stress response. Among them, Metallothionein 2A (MT2A) was screened out with high affinity. Subsequent surface plasmon resonance (SPR) experiments confirmed that ART directly binds to the MT2A protein with a dissociation constant (K_D_) of 5.38 µM. Further functional experiments demonstrated that ART treatment significantly upregulated the protein expression level of MT2A in astrocytes.

#### 4.1.2. Reducing Intracellular Cu^2+^ Levels and Ameliorating Dopaminergic Neuron Cuproptosis

MT2A is an important intracellular metal-chelating protein whose primary function is to bind excess metal ions (like copper, zinc) to mitigate their toxicity. By binding and upregulating MT2A, ART enhances the copper ion-chelating capacity of astrocytes. Experimental data showed that ART treatment effectively reduced the level of free Cu^2+^ within astrocytes. Since astrocytes play a key role in maintaining the homeostasis of the neuronal microenvironment, the reduction in their intracellular copper ion concentration indirectly decreases the copper burden in the environment surrounding neurons, thereby protecting dopaminergic neurons from cuproptosis. Rescue experiments confirmed this mechanism: when small interfering RNA (siRNA) was used to knock down MT2A in astrocytes, the ART-mediated effects of reducing intracellular Cu^2+^, alleviating dopaminergic neuron damage, and inhibiting cuproptosis were all abolished. Additionally, point mutation experiments identified lysine-31 (Lys-31) of the MT2A protein as a critical site for ART’s function, as mutating this site weakened the protective effect of ART [26].

#### 4.1.3. Regulating Cuproptosis-Related Proteins (FDX1, Lip-DLAT, CTR1)

By regulating intracellular copper homeostasis via MT2A, ART further influences the expression of key proteins in the core cuproptosis pathway. Studies indicate that ART treatment can modulate the expression of several cuproptosis-related proteins, including downregulating the key initiator of cuproptosis, FDX1 (ferredoxin 1, responsible for reducing Cu^2+^ to the more toxic Cu^+^), and the copper uptake protein CTR1 (copper transporter 1); simultaneously, it reduces the abnormal aggregation of the lipoylated protein DLAT (i.e., Lip-DLAT) in mitochondria [5,47,76]. These molecular alterations are consistent with—but do not by themselves constitute proof of—bona fide cuproptosis as strictly defined by Tsvetkov et al. (i.e., mitochondrial lipoylated protein aggregation, Fe-S cluster destabilization, and proteotoxic stress that is selectively rescued by copper chelators but not by inhibitors of apoptosis, ferroptosis, or necroptosis) [5,6]. In the PD study by Cheng et al., the anti-cuproptotic effect of ART was rigorously validated at the cell-death level: MT2A knockdown abolished ART-mediated neuroprotection, Cu^2+^ reduction, and the reversal of cuproptosis-associated protein changes [26]. Thus, the PD context represents the only currently validated instance where ART’s modulation of cuproptosis-associated proteins translates into demonstrable protection against cuproptosis cell death; in all other reported systems, ART’s effects are limited to cuproptosis-associated molecular phenotypes.

In summary, the novel mechanism by which ART exerts neuroprotective effects in Parkinson’s disease models involves its direct binding and upregulation of MT2A in astrocytes, enhancing cellular copper ion chelation, lowering intracellular free copper levels, and subsequently regulating the expression of key cuproptosis proteins like FDX1, CTR1, and Lip-DLAT, ultimately inhibiting cuproptosis in dopaminergic neurons. This finding identifies a novel therapeutic avenue for PD and broadens the scope of ART’s utility in modulating metal-dependent cell death.

#### 4.1.4. Context-Dependent GSH Dynamics: Reconciling Neuroprotection with Pro-Oxidant Activity

It is critical to note that the neuroprotective mechanism of ART in Parkinson’s disease models operates through a pathway that is mechanistically distinct from its pro-ferroptotic action in cancer cells, thereby resolving the apparent paradox of GSH dynamics. In the PD context, ART’s primary action is the direct binding and upregulation of MT2A in astrocytes (Section 4.1.1). This MT2A-mediated copper chelation pathway is functionally independent of the System Xc^−^/GSH/GPX4 axis. Mechanistically, this dichotomy arises because astrocytes exhibit a fundamentally different metabolic profile from cancer cells: they possess a lower labile iron pool (LIP) and a higher basal antioxidant capacity, which results in minimal cleavage of ART’s endoperoxide bridge via the Fenton reaction. Consequently, ART does not trigger significant ROS generation or GSH depletion in astrocytes. Instead, the upregulation of MT2A indirectly preserves the intracellular GSH pool by sequestering redox-active copper ions that would otherwise catalyze oxidative consumption of GSH. Furthermore, the protective effect on dopaminergic neurons is paracrine in nature: ART targets astrocytes, not neurons directly. Thus, neuronal GSH levels are preserved as a secondary consequence of reduced copper toxicity in the microenvironment. This cell-type-specific divergence in GSH kinetics—depletion in high-iron, high-xCT tumors versus preservation in low-iron, MT2A-upregulated astrocytes—is the key to reconciling ART’s dual role as a ferroptosis inducer in oncology and a cuproptosis inhibitor in neurodegeneration.

### 4.2. Potential Association of ART with Cuproptosis in Cancer Therapy

In contrast to its inhibitory effect on cuproptosis in PD models, ART is frequently paired with copper-based nanomaterials in cancer therapy to synergistically induce cuproptosis alongside its intrinsic ferroptosis-inducing activity. This context-dependent synergy (rather than standalone bidirectional regulation of cuproptosis by ART) highlights the disease-specificity of ART’s effects: standalone ART only inhibits cuproptosis in PD astrocytes, while pro-cuproptosis effects require exogenous copper supply that bypasses the low labile copper pool of tumors. Within the tumor microenvironment, ART is often designed for combination with copper-based materials or utilizes its own pharmacological properties to provide novel strategies for inducing tumor cell cuproptosis.

#### 4.2.1. Synergistic Induction of Cuproptosis with Copper-Based Nanomaterials (e.g., Cu-Fe_3_O_4_ Nanocluster Hydrogel)

Combining ART with copper-based nanomaterials to construct synergistic platforms is a strategy to enhance cuproptosis induction in tumors. For example, an injectable in situ hydrogel based on Cu-Fe_3_O_4_ nanoclusters (NCs), ART, and alginate (NCs-AS-ALG) was developed for synergistic treatment of osteosarcoma [67]. The Cu-Fe_3_O_4_ nanoclusters possess peroxidase, catalase, and glutathione peroxidase-mimetic activities, generating ROS in the tumor microenvironment. Fe^2+^/Fe^3+^ and Cu^2+^ ions released from the nanoclusters under acidic conditions undergo Fenton-like reactions with ART’s endoperoxide bridge, generating highly reactive carbon radicals and amplifying oxidative stress. This platform induces ferroptosis via ART-driven iron accumulation and GSH depletion, while Cu-Fe_3_O_4_-released Cu^2+^ activates cuproptosis via mitochondrial lipoylated proteins (e.g., DLAT). ART’s GSH depletion weakens Cu^2+^ chelation, lowering the cuproptosis threshold. Notably, cuproptosis is primarily driven by nanomaterial-derived copper, not ART alone.

Similar synergistic strategies are seen in other nano-formulations. A core–shell structured porous organic polymer hybrid nanoparticle (Cu/ART@Hpy) was designed for sonodynamic-enhanced cuproptosis therapy [77]. This nanosystem achieves pH-responsive release of Cu^2+^ in the acidic tumor microenvironment, while the loaded ART, activated by ultrasound, acts as a sonosensitizer to generate ROS and massively deplete intracellular GSH. The depletion of GSH weakens its ability to chelate copper ions, leading to a significant increase in Cu^2+^ accumulation within mitochondria, thereby effectively inducing cuproptosis. This design combines the sonodynamic effect of ART with the direct toxicity of copper ions, achieving multiple disruptions to the redox homeostasis of tumor cells, providing a new paradigm for combination therapy based on cuproptosis.

This Cu–Fe synergistic logic parallels the nano-ionophore architectures reviewed by Tolbatov and Marrone [27], where bimetallic Fe/Cu frameworks (e.g., CuFeGA MPNs, HaMOF) and targeted ionophores (e.g., RNP@Cu_2_O@SPF silencing ATP7A) are deployed to co-activate ferroptosis and cuproptosis in tumors. In our context, however, the ART-loaded CS@ART nanoparticles (Section 6.5.2) invert the therapeutic goal: rather than amplifying metal-driven RCD, the system exploits ART’s intrinsic MT2A-upregulating activity to suppress cuproptosis in the PD microenvironment, illustrating how the same Fe/Cu nano-synergy can be repurposed from oncology to neurodegeneration depending on the payload’s dominant molecular engagement. It is critical to emphasize that in these nanoplatforms, cuproptosis is primarily driven by nanomaterial-released Cu^2+^, not by ART alone. ART’s role is confined to GSH depletion and ROS amplification, which lower the cuproptosis threshold. Thus, labeling ART as a “cuproptosis inducer” in cancer would be misleading; a more accurate descriptor is “cuproptosis sensitizer” when co-delivered with exogenous copper sources.

#### 4.2.2. Indirectly Enhancing Copper Sensitivity by Depleting GSH

ART can effectively weaken the antioxidant defenses of tumor cells by depleting intracellular GSH, thereby indirectly sensitizing cells to copper-dependent cell death with cuproptosis-like features. The highly expressed GSH in tumor cells is a key barrier against copper toxicity, as it strongly chelates copper ions, preventing their accumulation within mitochondria and the triggering of the death program. ART and its active metabolite DHA deplete GSH, primarily through ROS generated from endoperoxide bridge cleavage, disrupting redox balance and reducing metal ion buffering capacity, allowing free copper ions to reach toxic thresholds. It should be noted that current evidence for ART’s engagement with cuproptosis predominantly demonstrates cuproptosis-associated molecular alterations—including modulation of FDX1, CTR1, and lipoylated DLAT (Lip-DLAT) expression, as well as reduction in intracellular Cu^2+^ levels—rather than direct proof of bona fide cuproptosis as strictly defined by Tsvetkov et al. (i.e., mitochondrial lipoylated protein aggregation, Fe-S cluster destabilization, and proteotoxic stress that is selectively rescued by copper chelators but not by inhibitors of apoptosis, ferroptosis, or necroptosis) [6,9]. In copper-based nanoplatforms, cuproptosis cell death is primarily driven by nanomaterial-released Cu^2+^, and ART’s contribution is confined to GSH depletion and ROS amplification that lower the cuproptosis threshold—a ‘cuproptosis sensitizer’ role rather than a ‘cuproptosis inducer’ role. Consequently, the term ‘cuproptosis’ as applied to ART in this review denotes a cuproptosis-associated or cuproptosis-prone state, unless explicitly supported by direct cell-death-level evidence. This evidence-based qualification is particularly critical given that ART alone—without exogenous copper supplementation—has not been demonstrated to induce cuproptosis cell death in any cancer cell line to date. In advanced nanotherapeutic platforms, ART’s GSH-depleting ability is amplified. In the Cu/ART@Hpy nanoparticle system, ultrasound-excited ART generates ROS, massively depleting GSH and creating a GSH-deficient microenvironment [77]. This allows co-delivered Cu^2+^ to accumulate in mitochondria and induce cuproptosis. Another strategy uses a nanoreactor (A@CuCD) co-assembled from copper nanoclusters and ART, encapsulated within γ-cyclodextrin (γ-CD) [78]. γ-CD disrupts cell membrane lipid rafts, downregulates PD-L1, and enhances cuproptosis sensitivity; ART synergistically promotes immunogenic cell death (ICD) and tumor microenvironment remodeling.

**Figure 5:**
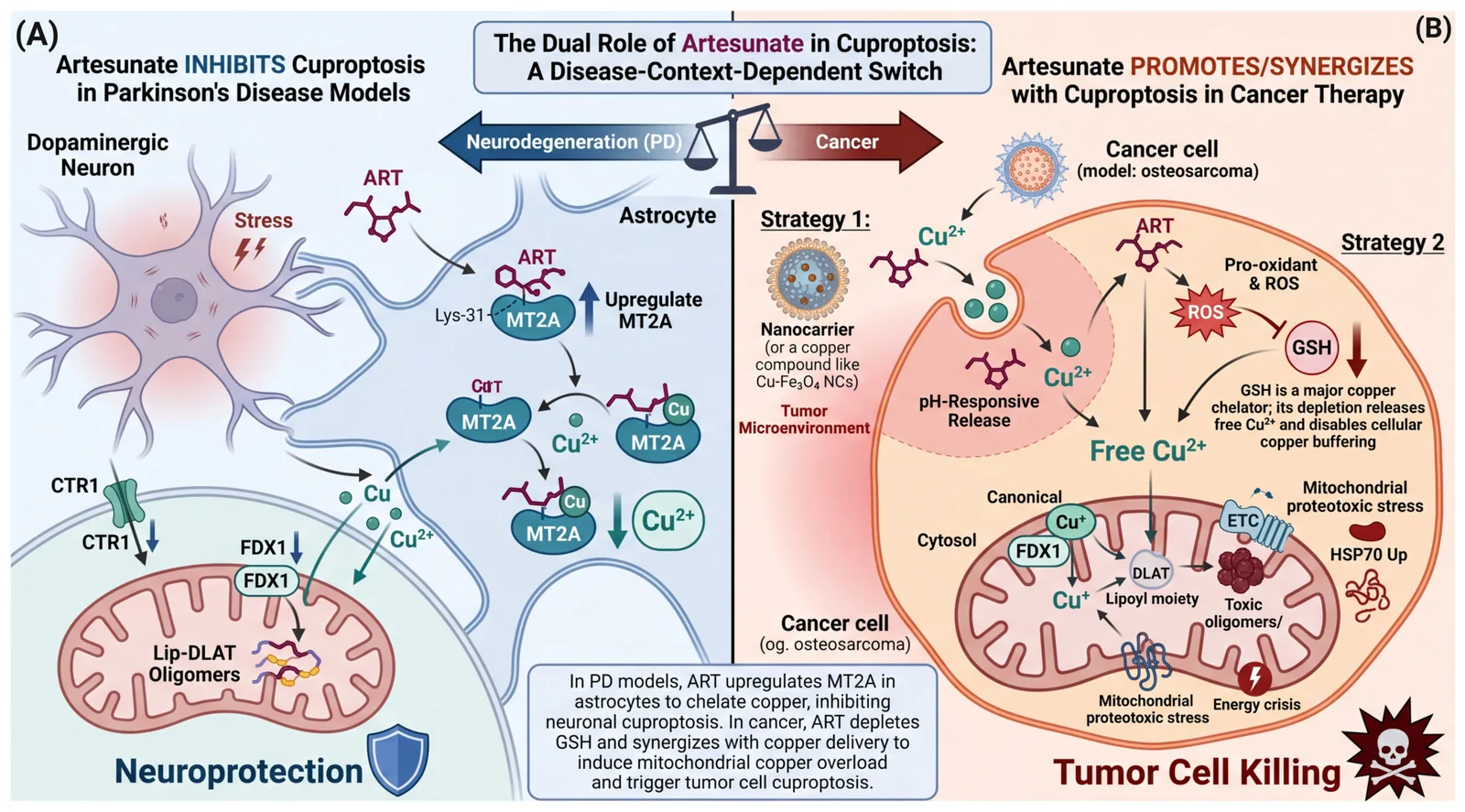
Context-Specific Associations Between ART and Cuproptosis: PD-Restricted Anti-Cuproptosis as Standalone Effect, and Pro-Cuproptosis Only in Copper Nanoplatform Combination. References: [26,67,77,78]. (**A**) Artesunate inhibits cuproptosis in Parkinson’s disease models via binding and upregulating MT2A in astrocytes. (**B**) Artesunate acts as a cuproptosis sensitizer in cancer therapy by depleting GSH and amplifying ROS. Note: the upward arrow (↑) indicates promotion or increase, the downward arrow (↓) indicates inhibition or decrease, and the T-bar lines indicate inhibition.

In summary, within cancer therapy, ART’s association with cuproptosis involves two strategies (Figure 5): first, as a component of copper-based nanomaterial platforms that simultaneously induce oxidative stress, ferroptosis, and cuproptosis; second, utilizing its ROS-generating ability to deplete GSH, disrupting redox homeostasis and enhancing cuproptosis. These strategies support the development of cuproptosis-based anti-tumor therapies. Key studies on ART-regulated cuproptosis, including the PD-specific anti-cuproptosis mechanism and nanoplatform-based pro-cuproptosis synergy, are summarized in Table 2.

## 5. Crosstalk Between Ferroptosis and Cuproptosis and the Synergistic Potential of ART

**Figure 6:**
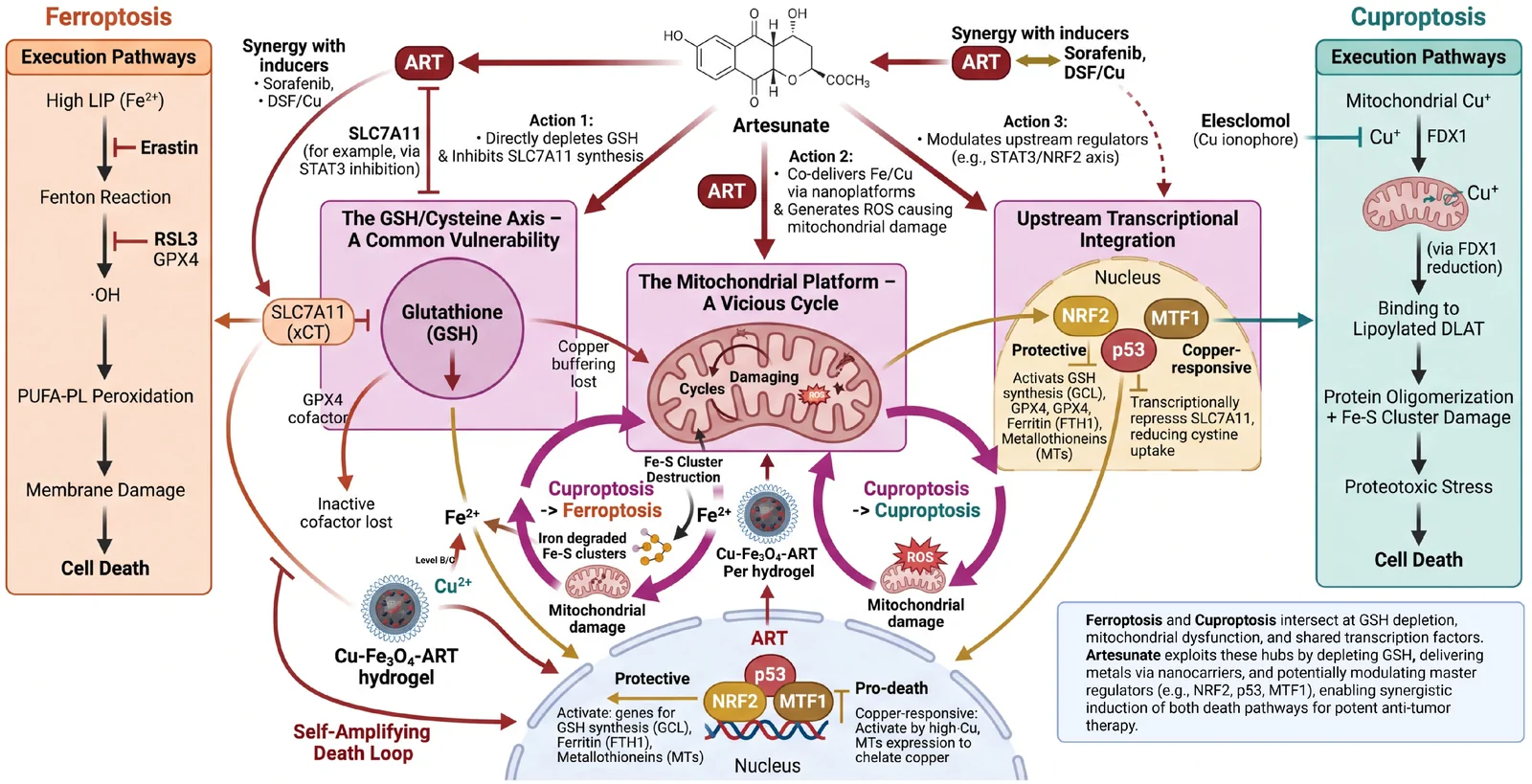
The Crosstalk Network Between Ferroptosis and Cuproptosis and the Synergistic Therapeutic Strategy Enabled by ART. Note: The diagram illustrates shared metabolic hubs (GSH/cysteine axis, mitochondria, ROS burst), metal homeostasis crosstalk (Cu^2+^-mediated ferritinophagy (hypothetical, Level B/C); GPX4 degradation; Fe-mediated copper transporter regulation), and overlapping upstream regulators (NRF2, p53, MTF1). References: [26,52,53,67,79]. The T-bar lines indicate inhibition.

Although ferroptosis and cuproptosis are driven by different metal ions (iron vs. copper) and involve distinct core execution proteins (e.g., GPX4 vs. lipoylated DLAT), research reveals crosstalk between them at the levels of metabolic regulation networks and cellular organelles. This interconnectedness provides a rationale for strategies targeting both pathways. ART acts on these shared hubs (Figure 6).

Interactions are classified into three evidence levels: Level A (experimentally confirmed in ART context), Level B (inferred from general cell biology, not yet tested with ART), and Level C (hypothetical, lacking direct evidence in any system). Each interaction below is annotated with its evidence level. A comprehensive summary is provided in Table 1.

### 5.1. Shared and Amplified Metabolic Hubs

#### 5.1.1. The GSH/Cysteine Axis: Shared Depletion Weakens Cellular Defenses

GSH is a shared node: it is a cofactor for GPX4 in ferroptosis and a chelator of Cu^+^ in cuproptosis [60]. Any factor causing GSH depletion simultaneously weakens resistance to both death modalities, which—at the theoretical level—would be expected to produce synergistic lethality (Level B, Table 1). It is critical to emphasize that this synergistic lethality is a theoretical inference for ART as a single agent; direct experimental demonstration of concomitant ferroptosis and cuproptosis execution by ART alone has not been reported in any cell type to date. However, it is critical to note that while GSH depletion is a necessary condition for cuproptosis, it is not sufficient to establish cuproptosis cell death—the latter requires mitochondrial copper accumulation to a threshold that triggers lipoylated protein aggregation and Fe-S cluster destabilization. ART alone has not been demonstrated to achieve this threshold in any cell type; the synergistic lethality predicted here remains a theoretical inference (Level B) rather than an experimentally confirmed outcome for ART as a single agent. This principle has been corroborated by non-ART systems such as dithiocarbamate–copper complexes (DSF/Cu), which induce both ferroptosis and cuproptosis via xCT stabilization and GSH depletion [1,80]. The precise magnitude of GSH depletion achieved by ART alone—as opposed to ART in combination with exogenous copper or nanoplatforms—has not been quantitatively benchmarked against canonical GSH-depleting agents (e.g., BSO, erastin). Yet, ART engages this shared GSH node through a distinct mechanism—its endoperoxide bridge generates ROS that deplete GSH through oxidation, rather than directly inhibiting System Xc- (this oxidative GSH depletion mechanism is inferred from non-ART systems and supported by ART studies measuring decreased GSH levels). As discussed in Section 4.1.4 and Section 5.4.1, the degree of GSH depletion is exquisitely sensitive to cellular iron load and the dominant stress-response pathway, explaining its divergent outcomes in cancer versus neurodegenerative contexts.

#### 5.1.2. Mitochondria as a Central Hub: A Vicious Cycle of Bidirectional Interaction

Mitochondria serve as a central platform for ferroptosis–cuproptosis crosstalk [81]. Cuproptosis exacerbates ferroptosis: copper overload degrades Fe–S cluster proteins, releasing free iron into the labile iron pool (LIP) and fueling Fenton-driven lipid peroxidation [47,60] (Level B, Table 1). Conversely, ferroptosis-induced mitochondrial damage (membrane densification, cristae disruption) impairs organelle homeostasis, rendering cells more sensitive to copper toxicity [81]. Furthermore, ferroptosis inducers like erastin can indirectly elevate mitochondrial copper levels by inhibiting respiration [1].

### 5.2. Crosstalk in Metal Homeostasis

Iron and copper have a bidirectional regulatory network governing intracellular homeostasis, forming a physiological basis for mutual influence between ferroptosis and cuproptosis.

#### 5.2.1. Copper Ions Regulate Ferritinophagy, Affecting the Iron Pool

Copper expands the LIP via ULK1/2-mediated NCOA4-dependent ferritinophagy and binds GPX4 at C107/C148, inducing TAX1BP1-dependent degradation (Level B) [53,82,83]. These findings suggest that copper overload may not only trigger cuproptosis but also prime cells for ferroptosis by simultaneously releasing iron and eliminating GPX4. However, this integrative hypothesis (Level C) has not been experimentally tested in any system, including ART. No direct primary study has demonstrated Cu^2+^-mediated NCOA4-dependent ferritinophagy in an ART-treated system; therefore, this pathway is indicated as hypothetical (Level B/C) in Figure 6.

#### 5.2.2. Iron Ions Regulate the Expression of Copper Transport Proteins

Iron status influences copper metabolism. Iron deficiency induces hypoxia-inducible factor-2α (HIF-2α), which cooperates with Sp1 to upregulate ATP7A (copper efflux) and metallothioneins (MTs) in intestinal epithelial cells (Level B) [84]. Iron deficiency may also upregulate copper import transporters (e.g., COPT2) via other transcription factors (Level B). This copper metabolic reprogramming alters intracellular copper levels, which may affect the cuproptosis threshold (Level C).

### 5.3. Overlap of Upstream Regulatory Factors

Ferroptosis and cuproptosis share several upstream regulators. NRF2 activates genes involved in GSH synthesis (GCL, GCLC), GPX4, ferritin (FTH1), ferroportin (FPN), and metallothioneins (MTs), thereby protecting against both death modalities (Level B) [50]. p53 transcriptionally represses SLC7A11, reducing GSH synthesis and promoting ferroptosis (Level B) [85,86]. Through GSH depletion, p53 may also sensitize cells to cuproptosis, but this remains speculative (Level C). MTF1 responds to copper stress by inducing MTs (Level B) [84]. Its role in ferroptosis is unknown (Level C). ART may influence these regulators; for example, ART inhibits STAT3, which cross-talks with NRF2 (Level B) [87,88,89]. Future studies should examine whether ART simultaneously modulates NRF2, p53, or MTF1 to achieve dual sensitization (Level C).

### 5.4. The Potential of ART as a Context-Dependent, Scenario-Specific Modulator of Both Pathways

The endoperoxide bridge of ART enables intervention in iron and copper metabolic networks. It can synergize with metal ions or death inducers to engage both ferroptosis and cuproptosis.

#### 5.4.1. Simultaneously Affecting Iron/Copper Metabolic Networks

A core action of ART is depleting GSH. GSH is a key molecule for resisting oxidative stress and metal toxicity; it is both a cofactor for GPX4 to scavenge lipid peroxides and a buffer for chelating free copper ions, preventing mitochondrial accumulation [90]. ART and its active metabolite DHA deplete GSH, primarily through ROS generated from endoperoxide bridge cleavage—the endoperoxide bridge reacts with intracellular free iron (and, to a lesser extent, copper ions) to generate ROS, which oxidize GSH to GSSG and overwhelm cellular antioxidant capacity. This dual suppression of the GSH pool weakens defenses against ferroptosis (via GPX4 inactivation) and cuproptosis (via reduced copper buffering), sensitizing tumor cells to both death modalities. Additionally, in ovarian cancer cells, ART downregulates SLC7A11 by inhibiting STAT3 signaling through the GP130/IL-6/STAT3/OTUB1 axis, reducing GSH synthesis at its source [21,63]; the relative contribution of endoperoxide-driven GSH oxidation versus transcriptional GSH synthesis blockade may vary across cell types and remains to be systematically dissected.

The GSH-depleting capacity of ART is context-dependent rather than universal. As detailed in Section 4.1.4, the extent of GSH depletion depends on two variables: (i) intracellular LIP level, which determines endoperoxide cleavage and ROS generation—the kinetic relationship between LIP, endoperoxide activation, and GSH oxidation rates has been primarily established in the antimalarial context and inferred for mammalian cancer cells; and (ii) cellular reliance on the System Xc^−^/GSH/GPX4 axis. In cancer cells with elevated LIP and xCT, ART triggers iron-catalyzed ROS production, SLC7A11 downregulation, and GSH depletion. In contrast, in PD astrocytes, LIP is low and the dominant pathway is MT2A-mediated copper chelation, which is GSH-independent. This differential engagement explains why ART can serve as a ferroptosis inducer in tumors and a cuproptosis inhibitor in the brain. Future studies should quantify cell-type-specific thresholds for ART-induced GSH depletion to predict its therapeutic window across disease contexts.

#### 5.4.2. Synergy with Metal Ions in Nanomedicine

Combining ART with metallic nanomaterials is a strategy to engage both pathways. A representative example is the Cu-Fe_3_O_4_ nanocluster–ART–alginate (NCs-AS-ALG) hydrogel, which releases Fe^2+^/Cu^2+^ to initiate ferroptosis and cuproptosis simultaneously (detailed in Section 4.2.1) [67]. The Cu-Fe_3_O_4_ nanoclusters possess peroxidase, catalase, and glutathione peroxidase-mimetic activities, generating ROS and reacting with ART’s endoperoxide bridge to produce carbon radicals. In the specific context of the Cu-Fe_3_O_4_ nanocluster platform, iron ions induce ferroptosis via GSH depletion and GPX4 inhibition, while copper ions trigger cuproptosis via DLAT aggregation and Fe-S cluster degradation. Within this engineered nanoplatform, Fe-S cluster degradation releases iron, exacerbating ferroptosis, and ferroptosis-induced mitochondrial damage enhances copper sensitivity, forming a self-reinforcing cycle [79] that has been demonstrated only under these supraphysiological, nanomaterial-delivered conditions. Extrapolation of this self-reinforcing cycle to ART as a single agent, or to physiological copper concentrations, is not supported by current evidence and remains a Level C hypothesis. However, the clinical translation of these multi-component nanoplatforms faces substantial hurdles, including scalable manufacturing, long-term metal bioaccumulation, and heterogeneous tumor targeting via the EPR effect—challenges that have not been addressed beyond proof-of-concept studies.

#### 5.4.3. Prospects for Synergistic Therapy in Combination with Ferroptosis/Cuproptosis Inducers

Combining ART with ferroptosis or cuproptosis inducers can overcome single-agent resistance. In hepatocellular carcinoma, ART combined with sorafenib (a System Xc^−^ inhibitor) synergistically promotes ferroptosis: sorafenib reduces cystine uptake and GSH synthesis, while ART amplifies oxidative stress and promotes ferritinophagy [21]. ART can also combine with disulfiram/copper (DSF/Cu), which blocks SLC7A11 degradation and depletes GSH, inducing both ferroptosis and cuproptosis; ART’s GSH-depleting ability adds to this effect. Furthermore, artemisinin–ferrocene conjugate (ART-Fc) provides local iron and generates ROS, achieving iron overload, ROS burst, and GSH depletion in a breast cancer model [91]. Most combination studies remain preclinical, and the optimal dosing sequence and ratio for maximizing synergy while minimizing toxicity have not been determined.

By depleting GSH and synergizing with metal ions via nanodelivery platforms, ART engages both ferroptosis and cuproptosis. Its combination strategies with existing inducers provide a direction for developing therapies based on metal-dependent cell death. Future research should explore ART’s regulatory role in more tumor models and optimize its nanoformulations and combination regimens to advance clinical translation.

## 6. Preclinical Therapeutic Implications and Translational Considerations

ART, an approved antimalarial drug, shows therapeutic potential beyond its original indication due to its ability to induce ferroptosis and regulate cuproptosis. To clearly distinguish the specific contributions of artesunate (ART) and avoid confounding single-agent effects with combination or nanoplatform studies, the following applications are categorized as (i) direct effects of ART as a single agent; (ii) ART acting as a sensitizer in copper-containing nanoplatforms, where cuproptosis is primarily driven by exogenous copper; (iii) findings obtained with DHA or other artemisinin derivatives; and (iv) proposed applications for which direct ART evidence is still lacking.

### 6.1. Direct Antitumor Effects of ART as a Single Agent

The anticancer activity of ART has been demonstrated in preclinical models of various solid and hematological tumors. A core mechanism appears to be the induction of ferroptosis, which may offer a preclinical basis for overcoming resistance to conventional chemotherapy in future clinical investigations. This section exclusively summarizes studies using ART as a single agent, without exogenous copper supplementation or nanocarrier co-delivery, to highlight ART’s standalone therapeutic contribution. Studies employing DHA, artemisinin, or other derivatives in lieu of ART are explicitly excluded from this single-agent classification, as these molecules exhibit quantitatively distinct potencies and kinetic profiles [73,74].

#### Monotherapy: Preclinical Evidence of ART-Induced Ferroptosis in Solid and Hematological Tumor Models

ART induces ferroptosis in multiple cancers through diverse, tumor-specific molecular mechanisms. In DLBCL, ART directly binds and inhibits PRDX1/2 [61]. In gastric cancer, it stabilizes TFRC by disrupting the TFRC–HSPA9 interaction, increasing iron uptake [64]. In ovarian cancer, ART suppresses SLC7A11 by binding to GP130 and disrupting the GP130/IL-6/STAT3/OTUB1 axis, leading to GSH depletion and GPX4 inactivation [63]. In bladder cancer, ART depletes GSH and inhibits GPX4 [92]. In colon cancer, ART reverses 5-FU resistance via Nrf2/GPX4 inhibition [93]. In osteosarcoma, ART activates NCOA4-mediated ferritinophagy to release free iron [65]. In hepatocellular carcinoma, ART upregulates WTAP-mediated m6A methylation of ATG5, enhancing autophagy-dependent ferritin degradation [66]. In activated hepatic stellate cells, ART promotes ROCK1/ATF3 axis-mediated SLC7A11 downregulation, exerting anti-fibrotic effects [62]. However, most studies employed ART concentrations exceeding clinically achievable plasma levels, and direct comparisons with canonical ferroptosis inducers (e.g., erastin, RSL3) are lacking, limiting quantitative benchmarking.

### 6.2. ART as a Sensitizer in Copper-Containing Nanoplatforms (Cuproptosis Driven by Exogenous Copper)

In contrast to its standalone anti-cuproptotic effect in Parkinson’s disease (Section 4.1), ART is paired with copper-based nanomaterials in cancer to synergistically induce cuproptosis. Crucially, in these platforms, cuproptosis is primarily driven by nanomaterial-released Cu^2+^, while ART acts as a sensitizer via GSH depletion and ROS amplification, rather than as a direct cuproptosis inducer. The term ‘cuproptosis inducer’ is therefore reserved for the nanoplatform as a whole, not for ART as a single agent. When describing ART’s standalone activity, the accurate descriptor is ‘modulator of cuproptosis-associated molecular pathways’—anti-cuproptotic in PD astrocytes via MT2A up-regulation, and cuproptosis-sensitizing in cancer via GSH depletion when exogenous copper is supplied. Representative examples include the Cu-Fe_3_O_4_ nanocluster–ART–alginate hydrogel (NCs-AS-ALG) [67] and Cu/ART@Hpy nanoparticles [77], which enable controlled release and simultaneous induction of ferroptosis and cuproptosis. In these platforms, ART’s GSH depletion weakens Cu^2+^ chelation, lowering the cuproptosis threshold. Another strategy uses a nanoreactor (A@CuCD) co-assembled from copper nanoclusters and ART [78]. It is critical to emphasize that ART alone does not induce cuproptosis in cancer. Labeling ART as a ‘cuproptosis inducer’ in cancer would be misleading—the nanoplatform as a whole is the inducer. A more accurate descriptor is ‘cuproptosis sensitizer’ when co-delivered with exogenous copper sources. The term ‘ART-based cuproptosis therapy’ is a misnomer when referring to ART alone; accurate terminology must explicitly acknowledge the engineered delivery vehicle and the exogenous copper source as the primary drivers of cuproptosis execution [75]. Clinical translation of these multi-component nanoplatforms faces substantial hurdles, including scalable manufacturing, long-term metal bioaccumulation, and heterogeneous tumor targeting via the EPR effect. Furthermore, ART’s rapid conversion to DHA and short pharmacokinetic half-life necessitate sustained-release formulations to maintain therapeutic concentrations—challenges that have not been addressed beyond proof-of-concept studies. Critically, while short-term preclinical toxicology suggests an acceptable safety margin at therapeutic doses, systematic chronic toxicity profiling of ART—particularly at the supra-therapeutic concentrations often required for ferroptosis induction—has not been performed and represents a prerequisite for clinical translation.

### 6.3. Combination Therapy with Chemotherapy, Radiotherapy, and Immunotherapy

Combination strategies may enhance ART efficacy in preclinical models, but the level of direct ART-specific evidence varies considerably. Co-administration with sorafenib has been shown to synergistically inhibit SLC7A11 and activate ferritinophagy in HCC models [21]. Combining ART with 5-FU reverses chemoresistance in colon cancer [93]. However, for several other combinations, direct ART-specific experimental evidence is currently lacking or limited. The combination with disulfiram/copper (DSF/Cu) has been proposed to achieve dual ferroptosis–cuproptosis activation via joint GSH depletion; however, this synergy has been primarily demonstrated with DSF/Cu alone, and direct evidence using ART (rather than its derivatives) in this combination is still lacking (Level C, hypothetical) [80]. Similarly, while ART acts as a radiosensitizer and can induce immunogenic cell death (ICD) in preclinical models, direct ART-specific evidence for synergy with immune checkpoint inhibitors or radiotherapy in the context of ferroptosis/cuproptosis requires further validation. Most combination studies remain preclinical, and the optimal dosing sequence and ratio for maximizing synergy while minimizing toxicity have not been determined.

### 6.4. Applications Based on DHA or Other Artemisinin Derivatives

Some mechanistic insights in this review derive from studies using DHA or other artemisinin derivatives rather than ART itself. These compounds share the artemisinin scaffold but differ in potency and cellular behavior; therefore, derivative-based findings are not extrapolated to ART as a single agent. ART-specific claims require ART-specific evidence. For example, DHA-induced ferritin degradation and the broader concept of artemisinin compounds amplifying iron overload via NCOA4-mediated ferritinophagy were established in studies that included both ART and DHA [70]; the relative contribution of each compound was not dissected. Additionally, an artemisinin–ferrocene conjugate (ART-Fc) provides local iron and generates ROS in a breast cancer model [91]. These findings are valuable but should not be directly attributed to ART as a single agent without confirmation. Future studies should verify whether ART alone replicates these effects to accurately define its therapeutic contribution.

### 6.5. Neurodegenerative Diseases and Other Non-Cancer Applications

Beyond cancer, ART exhibits therapeutic potential in neurodegenerative diseases and organ fibrosis, primarily through its anti-cuproptotic or anti-fibrotic mechanisms.

#### 6.5.1. Parkinson’s Disease: Protective Mechanism via Regulating MT2A

A study showed that ART protects dopaminergic neurons in a 6-OHDA-induced PD rat model by directly binding and upregulating astrocytic MT2A [26]. MT2A chelates excess Cu^2+^, reversing cuproptosis-related protein changes (FDX1, CTR1, Lip-DLAT). Knockdown of MT2A via AAV or siRNA abolished ART’s neuroprotective effects, confirming MT2A dependency. This establishes a “drug–astrocyte–MT2A–copper homeostasis” axis. However, this finding derives from a single study; replication in independent laboratories and additional PD models (e.g., α-synuclein-based) is needed before generalization. For CNS pharmacokinetics, ART is rapidly hydrolyzed to DHA, which shows better blood–brain barrier permeability [94,95]. Nevertheless, the short plasma half-life of DHA necessitates sustained-release or brain-targeted formulations.

#### 6.5.2. Ocular Fibrosis: A Non-Cancer Nanodelivery Application

For ocular diseases, a chitosan-based nanosystem (CS@ART) improved local sustained release and anti-fibrotic efficacy by suppressing fibroblast activation and inducing mitochondria-dependent ferroptosis [96]. Unlike the copper-containing nanoplatforms in Section 6.2, CS@ART does not involve exogenous copper or cuproptosis; it represents a non-cancer application of ART nanodelivery. In a rabbit glaucoma filtration surgery (GFS) model, a single subconjunctival injection of CS@ART significantly reduced collagen deposition and α-SMA/fibronectin expression at the surgical site, demonstrating superior anti-fibrotic effects compared to ART alone [96]. Mechanistically, CS@ART suppressed the Cyclin D1–CDK4/6, TGF-β1/SMAD, and PI3K/Akt signaling pathways while exhibiting marked anti-inflammatory effects [96]. This application highlights the potential of ART nanodelivery for non-cancer fibrotic diseases.

#### 6.5.3. Other Non-Cancer Applications and Hypothetical Extensions

ART also shows anti-fibrotic effects in liver fibrosis models via ROCK1/ATF3-mediated ferroptosis [62,97] and has been explored in cardiac fibrosis [98] and atherosclerosis [99,100]. For Alzheimer’s disease and Huntington’s disease, no direct evidence confirms ART regulates cuproptosis; these possibilities require experimental validation (Level C).

## 7. Current Challenges and Future Perspectives

Despite the preclinical therapeutic potential of ART in inducing ferroptosis and regulating cuproptosis, the path from basic research to clinical application remains nascent and faces several key challenges. First, the mechanistic understanding of ART’s effects is uneven across different cell death modalities. While evidence for ART’s anti-cuproptosis effect in PD via astrocytic MT2A is relatively clear [26], whether it directly regulates core cuproptosis execution proteins (e.g., lipoylated DLAT) in other disease models remains uncertain. Its direct interactions with key ferroptosis proteins (e.g., GPX4, ACSL4) also await elucidation. Second, ART’s effects exhibit significant cell-type and disease specificity. Resolving the GSH kinetic paradox—how ART depletes GSH to drive ferroptosis in tumors while preserving it for neuroprotection—is a mechanistic priority. Systematic quantification of cell-type-specific thresholds for endoperoxide bridge cleavage, LIP levels, and xCT expression is needed for predictive modeling of ART’s dual regulatory effects. For example, ART induces ferroptosis via PRDX1/2 in DLBCL [61] and via TFRC-HSPA9 [64] in gastric cancer, but inhibits cuproptosis in PD, reflecting dependence on the cell’s metabolic and redox state.

A related challenge lies in the evidentiary standard for asserting ‘ART-induced ferroptosis’. Definitive demonstration of ferroptotic cell death generally requires orthogonal pharmacological rescue—reversal of cell death by both a lipophilic antioxidant (ferrostatin-1 or liproxstatin-1) and an iron chelator (deferoxamine). Given that artesunate can concurrently induce apoptosis and autophagy, studies lacking such rescue are interpreted as demonstrating ferroptosis-associated phenotypic changes rather than unequivocally established ferroptotic cell death. Third, distinguishing the relative contributions of ferroptosis, cuproptosis, and other death modalities (e.g., apoptosis, necroptosis) to therapeutic outcomes in vivo remains a technical hurdle. Although systems like the Cu-Fe_3_O_4_ nanocluster hydrogel synchronously induce both deaths [67], multiple pathways are often interwoven under physiological conditions. Fourth, drug delivery and targeting remain challenges. ART undergoes rapid conversion to its active metabolite dihydroartemisinin (DHA) and has a short pharmacokinetic half-life (ART: 0.36–1.2 h; DHA: <1 h), which limits its bioavailability and therapeutic window. Additionally, ART lacks tissue or organelle specificity. Available safety data derive predominantly from acute/sub-chronic preclinical studies and short-term clinical use in malaria; the chronic toxicological profile of ART at the pharmacologically active concentrations required for ferroptosis induction (often exceeding clinically achieved plasma levels) remains inadequately characterized. Sophisticated nanoplatforms, such as Cu/ART@Hpy [77], have been engineered; however, their in vivo stability, long-term biosafety, and scalability require rigorous optimization. For CNS applications, optimizing BBB penetration and sustaining brain concentrations of ART/DHA through targeted nanocarriers are key translational steps. Fifth, clinical translation faces obstacles including determining the therapeutic window, systematically evaluating chronic safety—for which current evidence remains limited to sub-chronic preclinical toxicology studies and short-term clinical trials in cancer patients—especially for neurodegenerative indications requiring prolonged dosing, and developing biomarkers for real-time, non-invasive monitoring of ferroptosis/cuproptosis. As summarized in Table 1, most crosstalk interactions remain at Level B or C, underscoring the need for ART-specific validation before translational significance can be assessed. Sixth, regarding crosstalk between ferroptosis and cuproptosis: as systematically classified in Table 1, the vast majority of proposed ART-mediated dual-death-pathway interactions remain at Level B (inferred) or Level C (hypothetical). While these hypotheses provide a valuable roadmap for future experimental design, they should not be interpreted as established synergistic therapeutic mechanisms. We explicitly caution against over-interpreting the co-delivery nanoplatform studies as evidence that ART alone can coordinate ferroptosis and cuproptosis; such coordination has only been demonstrated in synthetic systems with supraphysiological metal concentrations. Future studies employing orthogonal pharmacological rescue (ferroptosis inhibitors + copper chelators) are essential to validate whether true dual-pathway synergy can be achieved with ART in physiological contexts. Seventh, chemical identity precision: future studies must explicitly designate whether observed effects derive from artesunate, DHA, the parent artemisinin, or synthetic derivatives, as these molecules exhibit quantitatively distinct ferroptosis-inducing potencies. Nanoplatform studies must further disaggregate contributions from the engineered metal source versus the artemisinin-class payload, to avoid over-attributing cuproptosis to ART itself.

Beyond these mechanistic gaps, several translational barriers merit attention. The short plasma half-life of DHA necessitates sustained-release formulations, yet dose-escalation studies defining the maximum tolerated dose for ferroptosis-based therapy are lacking, and available clinical safety data are restricted to short-term administration; whether such profiles extend to chronic dosing regimens required for neurodegenerative or anti-fibrotic indications remains undetermined. The selectivity of ART for cancer cells over normal tissues with high iron turnover (e.g., bone marrow, liver) has not been systematically quantified. For CNS applications, the absence of human BBB penetration data and the gap between nanocarrier designs and clinical strategies limit near-term translation.

Overcoming these challenges requires interdisciplinary approaches. Patient-derived organoids and single-cell multi-omics could map death signaling networks triggered by ART in different cell subpopulations. Stimulus-responsive nanodelivery systems could achieve controlled drug release at target sites. Blood- or imaging-based biomarkers (e.g., lipid peroxides, specific metabolites) are needed for patient stratification and therapeutic monitoring. Integration of multi-omics data using artificial intelligence may help predict optimal patient populations, effective drug combinations, and resistance mechanisms, facilitating clinical translation toward precision medicine based on metal-dependent cell death.

## 8. Conclusions

In summary, ART exhibits multi-target pharmacological properties extending beyond its antimalarial use. It regulates two metal-dependent cell death modalities—ferroptosis and cuproptosis—through independent or interwoven mechanisms. In cancer therapy, ART induces ferroptosis in various solid and hematological tumors by targeting PRDX1/2 and TFRC, or via GSH depletion and GPX4 inhibition. When combined with copper-based nanomaterials (e.g., Cu-Fe_3_O_4_ nanoclusters), it forms theranostic platforms that trigger both ferroptosis and cuproptosis in a reinforcing cycle. In neurodegenerative diseases such as Parkinson’s disease, ART upregulates astrocytic MT2A, chelating excess copper ions and inhibiting cuproptosis in dopaminergic neurons. These studies reveal crosstalk between ferroptosis and cuproptosis at metabolic hubs (e.g., GSH/cysteine axis), organelles (e.g., mitochondria), and upstream regulators (e.g., NRF2), providing a basis for designing ART-based synergistic therapies. However, direct evidence for ART’s regulation of cuproptosis outside the PD context remains limited, and most proposed crosstalk interactions under ART treatment await experimental validation. Future research into ART’s mechanisms using organoids, single-cell sequencing, and nanodelivery systems may advance its application in precision therapy for malignancies, neurodegenerative diseases, and organ fibrosis, facilitating a transition from drug repurposing to precision regulation.

## Figures and Tables

**Table 1:** Evidence Classification of Crosstalk Interactions Between Ferroptosis and Cuproptosis in the Context of Artesunate Treatment.

Crosstalk Node/Mechanism	Description	Evidence Level	Key References (ART-Specific)	Remarks
GSH depletion	ART/DHA depletes GSH via ROS generation and endoperoxide bridge cleavage	Level A	[21,61,62]	Directly demonstrated in multiple cancer models; GSH depletion confirmed by biochemical assays.
GPX4 inactivation	GSH depletion leads to GPX4 inactivation, triggering lipid peroxidation	Level A	[62,63]	Confirmed in ovarian cancer and hepatic stellate cells.
TFRC stabilization → iron overload	ART binds TFRC, blocks HSPA9 interaction, increases iron uptake	Level A	[64]	Validated in gastric cancer; rescue by TFRC knockdown.
Ferritinophagy (NCOA4)	ART upregulates NCOA4, promotes ferritin degradation, releases Fe^2+^	Level A	[65,66]	Shown in osteosarcoma and HCC.
PRDX1/2 inhibition	ART directly binds and inhibits PRDX1/2, causing ROS burst	Level A	[61]	Confirmed in DLBCL.
MT2A upregulation → Cu chelation	ART binds MT2A in astrocytes, upregulates its expression, chelates Cu^2+^	Level A	[26]	Only validated in PD models; rescue by MT2A siRNA.
Cu-Fe_3_O_4_ nanoplatform → dual death	ART + Cu-Fe_3_O_4_ NPs simultaneously induce ferroptosis and cuproptosis	Level A	[67]	ART acts as GSH depleter; cuproptosis driven by NP-derived Cu^2+^.
Fe-S cluster degradation → iron release	Cuproptosis causes Fe-S cluster degradation, releasing Fe^2+^ that amplifies ferroptosis	Level B	—	Demonstrated with elesclomol/DSF-Cu in general cuproptosis studies [5]; not yet tested with ART alone.
Mitochondrial dysfunction → ROS → ferroptosis sensitization	Cuproptosis-induced mitochondrial damage produces ROS that can potentiate ferroptosis	Level B	—	Well-established in general literature [1]; no direct ART-specific experiment.
Copper-induced GPX4 degradation	Cu^2+^ directly binds GPX4 and triggers its autophagic degradation (TAX1BP1)	Level B	—	Shown with CuCl_2_/elesclomol [53]; not tested with ART.
NRF2 regulation of both pathways	NRF2 transcriptionally activates GSH synthesis, MTs, and iron efflux genes	Level C	—	Hypothetical based on general NRF2 biology; ART’s effect on NRF2 in dual-death context unexplored.
p53/SLC7A11 axis	p53 represses SLC7A11, reducing GSH synthesis and sensitizing to both deaths	Level C	—	No direct evidence linking ART to p53-mediated cuproptosis sensitization.
MTF1/MTs regulation	MTF1 activates MT transcription in response to copper; ART may modulate this	Level C	—	Speculative; ART-MTF1 interaction not reported.

**Table 2:** Summary of Key Studies on ART-Regulated Ferroptosis and Cuproptosis.

Cancer Type	Model	ART Regimen	Key Target(s)	Death Type	Major Readout	Limitation
Diffuse large B-cell lymphoma	U2932, Riva, Oci-Ly8; normal B GM12878; BALB/c-nude s.c. xenograft	100 µM in vitro; 100 µL of 100 µM suspension i.g. q3d in vivo	PRDX1 (Gly4), PRDX2 (Arg7, Thr120)—direct binding (SM-pull down, CETSA, CD, mutagenesis); Kd 5–6 µM	Ferroptosis (major), weak apoptosis	PRDX1/2 KD → ROS ↑, MDA ↑, Fer-1 rescues; PRDX2 OE rescues; tumor weight ↓, ALT/AST ↓ in vivo	PRDX1 OE failed; IC_50_ not explicitly given in text
Osteosarcoma	MG-63, U2OS; SD rat tibial orthotopic	Cu-Fe_3_O_4_ NCs (PVP, ~150 nm) + artesunate (AS) loaded in ALG-Ca^2+^ hydrogel; in vitro AS 100 µM + NCs 100 µg/mL; single local implantation in vivo	Ferroptosis: GPX4 ↓, GSH ↓ (NCs POD/CAT/GSH-Px + AS carbon radical); Cuproptosis: NCs-Cu → DLAT oligomerization, FDX1	Ferroptosis (major) + Cuproptosis (minor, driven by NCs-Cu)	NCs triple enzyme activity at pH 6.5; synergistic killing (CI < 1); orthotopic tumor regression; Fer-1 + TTM partial rescue	Cuproptosis strictly from NCs-Cu, not from AS monomer; relies on local implantation
Liver fibrosis (hepatic stellate cells)	Human LX2, primary HSC; LO2 control; CCl_4_-induced ICR mice	5–20 µM in vitro; 5/10/20 mg/kg i.p. qd in vivo	ROCK1 (ubiquitin-proteasome degradation) → ATF3 (Thr162 dephosphorylation) → nuclear ATF3 → SLC7A11 ↓	Ferroptosis (major)	CHX + MG132 confirm proteasomal degradation; VA-Lip-Atf3-shRNA/VA-Lip-Rock1-plasmid in vivo HSC-targeted closure; collagen ↓	No commercial p-ATF3-Thr162 antibody; only indirect evidence via MPM-2 + T162D mutant
Gastric cancer	AGS, SGC-7901, HGC27, MGC-803, MKN45; normal GES-1, L02, MRC-5; BALB/c-nude s.c. xenograft	IC_50_: MGC-803 6.59, MKN45 9.04, AGS 96.11 µM; in vivo 50/100/200 mg/kg i.p. q.o.d.	TFRC (Lys10, Lys36, Glu147, PA domain 223–569)—direct binding (SPR Kd); HSPA9 indirectly (ART competes for TFRC → blocks HSPA9 → TFRC rerouted to RAB11 recycling)	Ferroptosis (major), weak apoptosis	Five-line IC_50_ correlates with baseline TFRC; shTFRC in vivo rescue; well tolerated up to 200 mg/kg	Whether HSPA9 acts as E3 adaptor unclear; TFRC-low sublines (AGS) insufficient for monotherapy
Parkinson’s disease	6-OHDA male SD rats; MN9D neurons, BV2 microglia, U118MG astrocytes; transwell co-culture	0.001–0.1 µM in vitro; 10/30 mg/kg p.o. ×14 d in vivo	MT2A (Lys31)—HuProt 20K screen → SPR Kd = 5.38 µM → astrocyte-specific; AS ↑ MT2A → Cu^2+^ chelation → FDX1 ↓, Lip-DLAT ↓, CTR1 ↑	Cuproptosis (inhibition, neuroprotective)	Co-culture: only neuron-astrocyte condition shows protection; AAV-MT2A KD + MT2A-K31R double rescue	Only males; no brain PK data; 0.1 µM in vitro contrasts sharply with tumor studies (5–100 µM)
Ovarian cancer	SKOV3, A2780, OVCAR3; OC-bearing mice	~10–40 µM in vitro; 5 mg/kg i.p. in vivo (tumor inhibition 82%/78%)	GP130 (Lys250)—BRET, CETSA, MD, mutagenesis → IL-6/STAT3 ↓ → OTUB1 ↓ → SLC7A11 deubiquitination loss	Ferroptosis (major)	Lowest effective single-agent dose in Table 2; STAT3 → OTUB1 confirmed by dual luciferase + ChIP; OTUB1–SLC7A11 co-IP	In vivo model details not specified; GPX4/iron pool not measured; PK data absent
Colon cancer (5-FU resistant)	HT-29/5-FU resistant line; parental HT-29 control	Single-agent IC_50_ > 100 µM; 40 µM + 5-FU 20 µg/mL → 5-FU IC_50_ 91.28 → 31.13 µg/mL (2.9-fold reversal)	Nrf2 ↓ → GPX4 ↓ (no SLC7A11/Fe^2+^/FTH1 measured)	Ferroptosis (major, Fer-1 rescue)	40 µM alone weak, combination restores sensitivity; colony ↓, ROS ↑, MDA ↑; Fer-1 reverses Nrf2/GPX4	No in vivo validation (acknowledged by authors); short mechanistic axis
Bladder cancer	T24; no in vivo	24 h IC_50_ 176 µM, 48 h 121 µM; main use 200 µM; DFOM/Z-VAD/CQ/Nec-1 quadruple inhibitor screen	GSH ↓, GPX4 ↓, FTH1 ↓; Nrf2 ↑ (compensatory resistance, reversible by DFOM)	Ferroptosis (major but non-exclusive; apoptosis, autophagy, necrosis also involved)	All four inhibitors partially rescue (DFOM slightly stronger); TEM typical ferroptosis mitochondria; Fe^2+^ 0.056 → 0.099 nmol/10^6^; previous lower ART concentrations 10 µM ART no ferro → this study uses 200 µM	Pure in vitro; Nrf2 direction opposite (compensatory); highest concentration in Table 2
Hepatocellular carcinoma (HCC)	HCC cell lines + orthotopic/xenograft (full text pending)	25–100 µM (abstract)	WTAP → m^6^A → ATG5 ↑ → ferritinophagy (NCOA4) → Fe^2+^ release	Ferroptosis (major)	m^6^A-autophagy-iron release triad; same team as #3 but HSC (anti-fibrosis) vs. HCC (cytotoxic)	Based on abstract; full text needed for target-level evidence
Breast cancer (ART-Fc conjugate)	BC cell lines + xenograft (full text pending)	ART-Fc conjugate (abstract)	GSH ↓, GPX4 ↓ (enhanced delivery via Fc conjugation)	Ferroptosis (major)	Only Fc-conjugate case in Table 2; parallels #2 (hydrogel NP) and #3 (VA-Lip) as “ART delivery modification” examples	Based on abstract; full text needed for detailed readouts

Note: the upward arrow (↑) indicates promotion or increase, the downward arrow (↓) indicates inhibition or decrease, and the right arrow (→) indicates induction or causal relationship. #2 refers to row 2 of Table 2, #3 refers to row 3 of Table 2, corresponding to the entry on Liver fibrosis (hepatic stellate cells).

## Data Availability

No new data were created or analyzed in this study. Data sharing is not applicable to this article.

## Abbreviations

The following abbreviations are used in this manuscript:

4-HNE	4-Hydroxynonenal
5-FU	5-Fluorouracil
ACSL4	Acyl-CoA Synthetase Long-Chain Family Member 4
ARE	Antioxidant Response Element
ART	Artesunate
ART-Fc	Artesunate–Ferrocene Conjugate
ATP7A/B	ATPase Copper Transporting Alpha/Beta
AAV	Adeno-Associated Virus
ATF3	Activating Transcription Factor 3
BBB	Blood–Brain Barrier
BSO	Buthionine Sulfoximine
DHA	Dihydroartemisinin
DHODH	Dihydroorotate Dehydrogenase
DLAT	Dihydrolipoamide S-Acetyltransferase
DLBCL	Diffuse Large B-Cell Lymphoma
EPR	Enhanced Permeability and Retention effect
ETC	Electron Transport Chain
Fe-S	Iron–Sulfur Cluster
FDX1	Ferredoxin 1
FSP1	Ferroptosis Suppressor Protein 1
FTH1	Ferritin Heavy Chain 1
GCH1	GTP Cyclohydrolase 1
GP130	Glycoprotein 130
GPX4	Glutathione Peroxidase 4
GSH	Glutathione
HaMOF	Hollow Metal–Organic Framework
HIF-2α	Hypoxia-Inducible Factor 2α
HSPA9	Heat Shock Protein Family A Member 9
HSC	Hepatic Stellate Cell
ICD	Immunogenic Cell Death
IRE	Iron-Responsive Element
IRP	Iron-Regulatory Protein
KD	Dissociation Constant
LIAS	Lipoyl synthase
Lip-DLAT	Lipoylated DLAT
LIPT1	Lipoyltransferase 1
LIP	Labile Iron Pool
LPCAT3	Lysophosphatidylcholine Acyltransferase 3
m6A	N6-Methyladenosine
MDA	Malondialdehyde
MD2	Myeloid Differentiation Factor 2
MTF1	Metal Regulatory Transcription Factor 1
MTs	Metallothioneins
MT2A	Metallothionein 2A
NCOA4	Nuclear Receptor Coactivator 4
NRF2	Nuclear Factor Erythroid 2-Related Factor 2
OXPHOS	Oxidative Phosphorylation
OTUB1	OTU Deubiquitinase 1
PCD	Programmed Cell Death
PD	Parkinson’s Disease
PD-L1	Programmed Death-Ligand 1
PRDX1/2	Peroxiredoxin 1/2
ROCK1	Rho-Associated Coiled-Coil Containing Protein Kinase 1
ROS	Reactive Oxygen Species
SLC7A11	Solute Carrier Family 7 Member 11
SNpc	Substantia Nigra Pars Compacta
Sp1	Specificity Protein 1
SPR	Surface Plasmon Resonance
STAT3	Signal Transducer and Activator of Transcription 3
TAX1BP1	Tax1 Binding Protein 1
TCA	Tricarboxylic Acid Cycle
TFRC	Transferrin Receptor
TLR4	Toll-Like Receptor 4
ULK1/2	Unc-51 Like Autophagy Activating Kinase 1/2
ZnT1	Zinc Transporter 1
6-OHDA	6-Hydroxydopamine
DSF/Cu	Disulfiram/Copper
